# Outcomes of a Comprehensive Mobile Smoking Cessation Program With Nicotine Replacement Therapy in Adult Smokers: Pilot Randomized Controlled Trial

**DOI:** 10.2196/41658

**Published:** 2022-11-24

**Authors:** Jennifer D Marler, Craig A Fujii, MacKenzie T Utley, Daniel J Balbierz, Joseph A Galanko, David S Utley

**Affiliations:** 1 Pivot Health Technologies Inc. San Carlos, CA United States; 2 Department of Pediatrics University of North Carolina Chapel Hill, NC United States

**Keywords:** smoking cessation, digital health, smartphone, digital sensor, carbon monoxide, breath sensor, biofeedback, mobile apps, health promotion, app, mobile phone

## Abstract

**Background:**

Cigarette smoking remains the leading cause of preventable illness and death, underscoring ongoing need for evidence-based solutions. Pivot, a US Clinical Practice Guideline–based mobile smoking cessation program, comprises a personal carbon monoxide breath sensor; a smartphone app; in-app, text-based human-provided coaching; nicotine replacement therapy; and a moderated web-based community. Promising Pivot cohort studies have established the foundation for comparative assessment.

**Objective:**

This study aimed to compare engagement, retention, attitudes toward quitting smoking, smoking behavior, and participant feedback between Pivot and QuitGuide, a US Clinical Practice Guideline–based smoking cessation smartphone app from the National Cancer Institute.

**Methods:**

In this remote pilot randomized controlled trial, cigarette smokers in the United States were recruited on the web and randomized to Pivot or QuitGuide. Participants were offered 12 weeks of free nicotine replacement therapy. Data were self-reported via weekly web-based questionnaires for 12 weeks and at 26 weeks. Outcomes included engagement and retention, attitudes toward quitting smoking, smoking behavior, and participant feedback. The primary outcome was self-reported app openings at 12 weeks. Cessation outcomes included self-reported 7- and 30-day point prevalence abstinence (PPA), abstinence from all tobacco products, and continuous abstinence at 12 and 26 weeks. PPA and continuous abstinence were biovalidated via breath carbon monoxide samples.

**Results:**

Participants comprised 188 smokers (94 Pivot and 94 QuitGuide): mean age 46.4 (SD 9.2) years, 104 (55.3%) women, 128 (68.1%) White individuals, and mean cigarettes per day 17.6 (SD 9.0). Engagement via mean “total app openings through 12 weeks” (primary outcome) was Pivot, 157.9 (SD 210.6) versus QuitGuide, 86.5 (SD 66.3; *P*<.001). Self-reported 7-day PPA at 12 and 26 weeks was Pivot, 35% (33/94) versus QuitGuide, 28% (26/94; intention to treat [ITT]: *P*=.28) and Pivot, 36% (34/94) versus QuitGuide, 27% (25/94; ITT: *P*=.12), respectively. Self-reported 30-day PPA at 12 and 26 weeks was Pivot, 29% (27/94) versus QuitGuide, 22% (21/94; ITT: *P*=.32) and Pivot, 32% (30/94) versus QuitGuide, 22% (21/94; ITT: *P*=.12), respectively. The biovalidated abstinence rate at 12 weeks was Pivot, 29% (27/94) versus QuitGuide, 13% (12/94; ITT: *P*=.008). Biovalidated continuous abstinence at 26 weeks was Pivot, 21% (20/94) versus QuitGuide, 10% (9/94; ITT: *P*=.03). Participant feedback, including ease of setup, impact on smoking, and likelihood of program recommendation were favorable for Pivot.

**Conclusions:**

In this randomized controlled trial comparing the app-based smoking cessation programs Pivot and QuitGuide, Pivot participants had higher engagement and biovalidated cessation rates and more favorable user feedback at 12 and 26 weeks. These findings support Pivot as an effective, durable mobile smoking cessation program.

**Trial Registration:**

ClinicalTrials.gov NCT04955639; https://clinicaltrials.gov/ct2/show/NCT04955639

## Introduction

### Background

Tobacco use is responsible for more than 8 million deaths around the world per year. On its own, smoking is a leading cause of preventable illness and death worldwide [1]. Despite this, most quit attempts are undertaken without assistance and are unsuccessful [2].

In recent years, mobile app–based programs for smoking cessation have become prevalent and show promise with greater accessibility than traditional face-to-face programs. A variety of these programs currently exist, but many lack evidence of their efficacy. A 2019 meta-analysis by Whittaker et al [3] analyzed 5 studies and found no evidence that smartphone app cessation programs improved smoking cessation outcomes when compared with lower-intensity cessation apps or minimal nonapp support (relative risk ratio [RR] 1.00, 95% CI 0.66-1.52; *I*^2^=59%). This finding was of low certainty, however, owing to inconsistencies and imprecision, highlighting the need for more randomized controlled trials (RCTs) of app-based cessation programs.

Bricker et al [4] compared 2 app-based cessation programs in a 2020 RCT. At 12 months, participants randomized to iCanQuit, an acceptance and commitment therapy (ACT)–based smoking cessation app, had 1.49 times higher odds of quitting smoking than participants randomized to QuitGuide, a US Clinical Practice Guideline (USCPG)–based smoking cessation app. Previously, in 2014, Bricker et al [5] ran a similar RCT comparing SmartQuit, another ACT-based smoking cessation app, with QuitGuide. At 2 months, 13% of SmartQuit and 8% of QuitGuide participants quit smoking (odds ratio [OR] 2.7, 95% CI 0.8-10.3). Another RCT, by BinDhim et al [6] in 2018, compared a smoking cessation decision-aid app with an information-only control app. At 6 months, 10.2% using the decision-aid app and 4.8% using the control self-reported continuous abstinence from smoking (RR 2.02, 95% CI 1.08-3.81).

More comprehensive programs with nicotine replacement therapy (NRT) and additional support have also been studied. In a 2020 RCT, Webb et al [7] compared a cognitive behavioral therapy (CBT)–based smoking cessation app with one-on-one coaching (Quit Genius) to Very Brief Advice. All participants had access to 3 months of NRT and a random half of each arm received a carbon monoxide (CO) breath sensor device. At 52 weeks, 34.7% (92/265) of the participants in the treatment arm achieved 7-day point prevalence abstinence (PPA) versus 29.4% (78/265) in the control (RR 1.20, 95% CI 0.94-1.54). The assignment of the CO breath sensor device, or lack thereof, did not significantly predict whether a participant achieved 7-day PPA [7]. Tweet2Quit, a program including an app, SMS text messages, and a Twitter group, was compared with a nonapp control in a 2016 RCT by Pechmann et al [8]. Both groups received 56 days of NRT patches, instruction to set a quit date, and referral to the National Cancer Institute’s smoking cessation website [9]. At 60 days, the Tweet2Quit arm had 40% smoking abstinence compared with 20% among controls.

Technology-enabled features of smoking cessation programs, including CO breath sensors, web-based communities, and SMS text messages–based coaching have been explored previously. In *The Tobacco Dependence Treatment Handbook: A Guide to Best Practices* [10] the authors reported that “providing individualized feedback about changes in personal levels of carbon monoxide before and after smoking is a powerful message that encourages individuals to make a quit attempt,” demonstrating the utility of CO monitors for smoking cessation. Beard and West [11] provided smokers not seeking out a quit smoking program with personal CO breath sensors for 6 weeks, with a goal to maintain their CO level <10 parts per million (ppm). Participants were not instructed to quit. The 10 participants used the CO monitors an average of 3 times a day, decreased their average daily cigarette consumption from 14.1 (SD 6.03) at baseline to 9.8 (SD 4.95; *P*=.04) during the 2 weeks of daily CO monitoring and to 9.5 (SD 5.50; *P*=.13) at the 6-week follow-up. At follow-up, 50% (5/10) of the participants had attempted to quit smoking and one successfully quit. Most (111/140, 79.3% of the responses) participants reported the CO monitor was helpful and that they felt as though the monitor (7/10, 70% of the participants) had reduced their cigarette consumption. Beard and West [11] concluded that the use of the CO monitors increased motivation to consider a quit attempt. A 2020 cohort study also assessed the use of a personal CO breath sensor, specifically the Pivot Breath Sensor, by 234 adult smokers. The sensor’s impact on attitudes toward quitting smoking and smoking behavior was investigated over 12 weeks. Participants in this study had a significant (*P*<.001) increase in motivation to quit smoking, 28.2% (66/234) made at least 1 quit attempt, and 38.5% (90/234) reduced the number of cigarettes smoked per day at 12 weeks [12].

Smoking cessation programs with web-based communities have also been studied. Graham et al [13] conducted a propensity score weighting of the iQUITT study, an RCT of telephone and internet treatment for smoking cessation, where the internet arm of the study included a large and well-established web-based community. Of the 492 participants assigned to the iQUITT study’s internet arm, 198 (40.2%) did not engage with the web-based community, 184 (37.4%) engaged both actively and passively, and 110 (22.4%) engaged only passively. At 3 months, Average Treatment Effects weighted abstinence rates were 4.2% for those who did not use the web-based community, 15.1% for those who used the web-based community passively, and 20.4% for those who used the web-based community both passively and actively. Users of the web-based community were also more likely to quit smoking than nonusers. Sadasivam et al [14] conducted a study testing the functions of Decide2Quit.org, a web-based tobacco intervention that contains a web-based community, SMS text messaging with tobacco treatment specialists, and other major functions to support tobacco cessation. In bivariate comparison among 204 smokers, the web-based community had a positive association with quit outcomes at 6 months and the highest differential in quit outcomes for those that used the function compared with other functions of the web-based quit program. SMS text messaging with tobacco treatment specialists was negatively associated with quit outcomes at 6 months; however, the authors suggest that these results could be confounded by those using the specialists as having the most difficulty in quitting smoking.

Studies focused on the impact of one-on-one text coaching or SMS text messaging with tobacco treatment specialists are limited. Sadasivam et al [15] conducted a secondary analysis of a web-based smoking cessation intervention that includes asynchronous messaging with trained tobacco treatment specialists. The goal of the study was to evaluate the association of this communication with smoking cessation during a period of 6 months. Of the 725 smokers in the study, 245 (33.8%) messaged a tobacco treatment specialist at least once. The amount of SMS text messaging with a tobacco treatment specialist had no association with cessation outcomes at 6 months, although the authors suggest low engagement or lack of power to be explanations for the lack of association found.

A cohort study of the Pivot program was published in 2021 (N=319). During the study, Pivot included a mobile app, a personal CO breath sensor, and text-based human-provided coaching. At 3 months after program completion (mean 7.2, SD 1.2 months after enrollment), 32% (intention to treat [ITT]) and 37.5% (completer) of the participants achieved 7-day PPA; 27.6% (ITT) and 32.4% (completer) reported 30-day PPA [16]. The Pivot program has since undergone updates and now includes access to NRT and a moderated web-based community. These changes, the need for long-term results for app-based cessation programs, and the ongoing need to assess the performance of Pivot within the context of current smoking cessation programs, warrant new investigation of the Pivot program.

### Objectives

The primary aim of the study was to compare user engagement and retention in the Pivot smoking cessation program to the current mobile standard of care. The secondary aims were to compare changes in attitudes toward quitting smoking, changes in smoking behavior, and feedback on the user experience.

## Methods

### Design

In this 2-arm, parallel-group, noncrossover, single-center RCT, participants were randomized to 1 of 2 app-based smoking cessation programs: QuitGuide (control) or Pivot (intervention). All participants had access to 12 weeks of free NRT. A total of 6 reminders to prompt use of the program were emailed to all participants every other week over the first 12 weeks of the study. User engagement and retention, attitudes toward quitting, smoking behavior, and participant feedback were compared between the 2 groups. Here, we report outcomes through 26 weeks, as data collection for the 1- and 2-year time points is ongoing. The study was performed remotely on an ambulatory basis.

### Ethics Approval

All participants provided electronic informed consent before participation. The study was reviewed and approved by Solutions IRB, LLC (protocol number 2021/04/38) and registered with ClinicalTrials.gov (NCT04955639).

### Participants

Eligibility criteria are presented in Textbox 1.

**Eligibility criteria.**

**Inclusion criteria**

- Aged ≥21 years of age
- Current daily cigarette smoker (≥5 cigarettes per day) for the past 12 months
- Plans to quit smoking in the next 30 days
- Resident of the United States
- Able to read and comprehend English
- Owns and uses a smartphone compatible with the study app (iPhone 5 and above, with operating system iOS 12 and above or Android 7.0 and above, with operating system Android 7.0 and above)
- Has daily internet access on smartphone
- Self-reported comfort with downloading and using smartphone apps

**Exclusion criteria**

- Pregnancy (self-reported)
- Health contraindications to nicotine replacement therapy use (irregular heartbeat, high blood pressure not controlled with medication, heart attack or stroke within the last 2 months, breastfeeding, skin allergies to adhesive tape or serious skin problems, stomach ulcers, or history of seizures)
- Using other smoking cessation support, including apps, or actively taking medication to quit smoking
- Daily marijuana use
- Residence with another study participant
- Immediate family member is a study participant
- Failure to provide contact information or verify email address
- Participation in a previous study sponsored by Pivot Health Technologies Inc. (formerly Carrot Inc)

### Recruitment

Participants were recruited in the United States through web media (Facebook and Google Ads). Potential participants were asked to provide contact information and answer questions on demographics (gender, age, employment status, location via city and state, and race and ethnicity), smartphone ownership, and smoking attitudes and behavior (Stage of Change and cigarettes per day [CPD]) using a web-based screening form. Study staff reviewed each web-based screening form.

Using nonproportional quota sampling, potential participants were called on a first-come-first-served basis, with the aim to enroll 40% to 60% men, no more than 50% of the participants from any decade-spanning age group (eg, 30-39 years of age), no more than 70% of the participants in the non-Hispanic White race category, and up to 20% not employed. The goals of these nonproportional quota sampling ranges were to ensure representation among men, racial and ethnic minorities, age groups, and individuals with varying socioeconomic status. Regarding the nonproportional quota sampling for employment, at the time of protocol design (March 2021 and April 2021), the unemployment rate in the United States was 6% [17]. Acknowledging a higher unemployment rate among people who smoke [18-21] and the desire to include individuals who either do not receive payment for their work or are not pursuing employment (stay-at-home parents, caretakers, students, or retired individuals), we sought to enroll up to 20% of participants who did not have compensated employment.

During the screening phone call, potential participants were asked questions to confirm study eligibility. During this call, study personnel informed the potential participant of the study details and answered any questions.

Potential eligible participants who wanted to proceed with the study were emailed an electronic Health Insurance Portability and Accountability Act authorization form and an electronic informed consent form, which they signed before participating in this study.

### Randomization and Blinding

Participants were randomly assigned in a computer-generated 1:1 ratio to either QuitGuide or Pivot using randomly permuted blocks of sizes 2 and 4. The allocation sequence was provided by the Study Randomizer (Phase Locked Software, 2017) application [22]. Participants were stratified by daily smoking frequency (≤14 vs ≥15 CPD), employment status (full-time or part-time employment vs not employed), race and ethnicity (minority race and ethnicity vs non-Hispanic White) and expected difficulty staying quit (scale 1-10; self-reported score of ≤5 vs ≥6). These 4 factors were chosen, as they have been associated with cessation outcomes in prior studies [16,23-28]. Researchers were blinded to treatment allocation until after randomization was performed.

### Intervention: Pivot

Pivot is a 12-month digital smoking cessation program based on the USCPG for tobacco use cessation. Pivot includes the Pivot Breath Sensor and Pivot app (Pivot Health Technologies Inc).

The Pivot Breath Sensor is a portable, personal mobile breath sensor that measures the level of CO in exhaled breath. The user submits a breath sample by exhaling into the sensor mouthpiece. The sensor displays the exhaled breath CO value in ppm to the user directly on the device. When paired to the user’s smartphone, the user’s CO values also populate the Pivot app, where they can be accessed by the user. Displayed CO values are color coded and categorized as most consistent with not smoking (green, 0-6 ppm), possibly smoking (orange, 7-9 ppm), or smoking (red, ≥10 ppm). There was no required use of the sensor; however, the participants were informed that suggested use of the sensor is 4 times per day, spread out over the course of the day, acknowledging they should use the sensor as it best fits with their lives. Users may use the sensor to link their smoking behavior and CO values and track their progress in reducing or quitting smoking.

The self-guided Pivot app leverages evidence-based principles and clinical best practices. This includes the USCPG-recommended 5 A’s (Ask, Advise, Assess, Assist, and Arrange), tailoring on readiness to quit [29], the provision of Food and Drug Administration (FDA)–approved NRT with accompanying education on use and adherence [29-31], the incorporation of effective methods for smoking cessation based on CBT and self-determination theory [32-34], and CBT-based counseling through a live, dedicated coach [29,33,35]. Pivot app functions include interactive educational activities, the ability to log cigarettes, set a quit date, create a quit plan, complete practice quits (1-24 hours in duration), play educational games, watch educational videos, interact with one’s dedicated human coach via in-app text messaging, view CO breath sample values and trends, learn about and then order NRT, access the moderated web-based Pivot community discussion forum, share goals and progress with the web-based Pivot community discussion forum or one’s social network via SMS text messaging or email, and complete daily check-ins after quit date.

The educational journey in the Pivot app comprises 4 tracts, Learn, Reduce, Prepare to Quit, and Maintain My Quit, and is designed to accommodate smokers along the spectrum of readiness to quit. Participants may choose to focus on building self-awareness and learn more about their smoking behavior, create and practice their plan to quit or reduce smoking, make a quit attempt, focus on staying quit, or any combination thereof. Accordingly, participants may navigate between tracts as desired to access content most relevant to their goals and needs.

Pivot users are assigned a human coach with whom they work one-on-one over the duration of their use of Pivot (up to 1 year). Communication between coach and Pivot user is via asynchronous in-app text messaging. Pivot coaches are tobacco treatment specialists. The coach reaches out periodically, approximately once per week, during the participant’s active use of Pivot. Participants may reach out to their coach whenever and however often they like.

Pivot users may access the moderated web-based discussion community through the Pivot app. The forum is moderated by a tobacco treatment specialist. The web-based community forum is a place to give and receive support and advice from others going through the Pivot program.

### Control: QuitGuide

QuitGuide is a product [9] of a smoking cessation resource created by the Tobacco Control Research Branch at the National Cancer Institute in collaboration with tobacco control professionals and smoking cessation experts and with input from ex-smokers [36]. A well-established smoking cessation app, QuitGuide, has been used in previous RCTs in which digital smoking cessation programs were compared [4,5]. The app focuses on helping users understand their smoking patterns and build the skills needed to become and stay smoke free [36]. Specifically, QuitGuide helps users to focus on motivations to quit; prepare to quit through developing a quit plan, identifying and planning how to address triggers and moods, teaching about FDA-approved smoking cessation medications, and identifying and providing access to social support; quit smoking by acknowledging user progress and teaching skills to address cravings; and stay quit by presenting tips and motivations to stay smoke free and address slips if they occur. QuitGuide app functions include educational reading activities, including focus on FDA-approved cessation medications and associated adherence. Additional QuitGuide app functions comprise tracking and reviewing cigarettes, moods, triggers, and cravings; setting tip message notifications for locations and times when one is prone to smoke; setting a quit date; creating a quit plan; completing journal entries; sharing goals and progress with one’s social network via SMS text messaging or email; accessing additional chat and phone support; and providing updates on quit status after quit date.

QuitGuide was used as the control for the following reasons: the content follows the USCPG for tobacco cessation; it is an app-based smoking cessation program, thereby enabling intrastudy comparison of same-modality interventions; the app is nonproprietary and is free to the public; and its use in previous well-designed RCTs [4,5] provides context and enables interstudy comparison to earlier data.

### Nicotine Replacement Therapy

Participants had access to free FDA-cleared over-the-counter NRT. Participants were provided with on-label information about the NRT and were able to order it on the web (QuitGuide) or in their study app (Pivot). The types of NRT offered included nicotine patches (7, 14, or 21 mg), nicotine gum (2 or 4 mg), and nicotine lozenges (2 or 4 mg). Participants could order patches, gum, or lozenges alone as monotherapy or patches with either gum or lozenges as combination therapy. Participants were able to order NRT every 2 weeks for up to a 12-week course over the first 12 months of the study. Engagement emails were sent to participants at weeks 1 and 3, reminding them of the availability of NRT and how to order it.

### Biovalidation

Biovalidation was sought at 12 and 26 weeks in individuals who reported 7-day or greater PPA on the associated questionnaire. A video call with study staff and the participant was scheduled within 7 days following the participant’s response to the associated questionnaire. At the beginning of each biovalidation visit, participants were asked their CPD, 7-day PPA status, and if they had smoked any other noncigarette (eg, pipes, cigars, or hookah) or combustible materials (eg, cloves or marijuana) over the previous 24 hours.

Participants who indicated they were not at least 7 days abstinent or that they smoke ≥1 CPD were not eligible to undergo further biovalidation testing during the visit. Participants who indicated they were at least 7 days abstinent and did not smoke cigarettes were eligible to proceed with the testing. Participants who indicated they had smoked any other combustible materials over the previous 24 hours were eligible to undergo biovalidation test at that same visit, with the possibility of scheduling a follow-up biovalidation test for the following day with instruction to not smoke the previously reported other combustible substance(s) over the intervening 24-hour period. If a participant was eligible for biovalidation and biovalidation was not achieved, the reason was noted (did not schedule or attend a biovalidation study visit, reported change in smoking status at outset of visit, participant’s breath CO sample was ≥10 ppm, etc).

Biovalidation was obtained through CO breath sampling. Participants in the intervention arm used their Pivot Breath Sensor for this test. Shortly before the visit, participants in the control arm were mailed a Pivot Breath Sensor limited to 10 breath samples. On the video call, participants held the breath sensor up to the screen immediately after completing the breath sample so that study staff could see and record the CO ppm measurement on the sensor screen. A CO value of <10 ppm was considered consistent with abstinence [37,38].

After their first biovalidation visit, participants in the control arm were instructed to not use the breath sensor beyond the visit and to place the sensor in a safe place to access for use at a future biovalidation visit should there be one. For subsequent biovalidation visits, participants used their existing breath sensor or were mailed a new one as needed.

### Outcomes and Measures

#### Baseline

The following variables were collected at baseline: demographic information (age, gender, race and ethnicity, household income, education, employment status, and smartphone type); smoking status; smoking history; Heaviness of Smoking Index [39]; success to quit (STQ; scale 1-10) and difficulty to stay quit (DTQ; scale 1-10) [40,41]; and Smoking Abstinence Self-efficacy Questionnaire (SASEQ)—a 6-item survey describing emotional or social situations for which smokers indicate on a 5-point Likert scale (0-4) whether they will be able to refrain from smoking, with total higher scores representing higher self-efficacy [42].

Study outcomes focused on 4 areas: user engagement and retention, attitudes toward quitting, smoking behavior, and participant feedback.

#### User Engagement and Retention

The preregistered primary outcome of the study was total app openings in Pivot versus QuitGuide at 12 weeks. Additional outcomes included the number of days and number of weeks with ≥1 app opening. App openings were self-reported weekly for the first 12 weeks of the study. Self-report of app use has been reported previously [5] and was necessary because automatic recording of this information was not enabled for QuitGuide.

#### Attitudes Toward Quitting Smoking

Measures reflecting attitudes toward quitting included the desire to quit (yes or no), STQ (scale 1-10) and DTQ (scale 1-10) [40,41], and SASEQ [42].

#### Smoking Behavior

Smoking behavior assessment comprised quit attempts, CPD (mean percentage change and the proportion of participants who reduced their CPD by ≥50% compared with baseline), smoking cessation via self-reported 7- and 30-day PPA and biochemically confirmed abstinence, continuous abstinence (self-report and biochemically confirmed), abstinence from all tobacco products (self-report), and the use of NRT.

Participants were considered to have made a quit attempt during the study if they answered ≥1 to the following question: “Since you began the study, how many times have you tried to quit smoking where you’ve gone at least 1 day without smoking a cigarette, even a single puff?” From this question, mean (SD) quit attempts per participant were quantified as well. Participants were considered to have achieved self-reported 7-day (30-day) PPA if they answered “no” to the following question: “In the last 7 (30) days, have you smoked any cigarettes, even a single puff?” Biochemically confirmed abstinence was defined as self-reporting 7-day abstinence and a breath CO sample <10 ppm at the associated biovalidation visit. Self-reported continuous abstinence was assessed at 26 weeks and was defined as self-report of 7-day (or greater) PPA at 12 weeks, self-report of 30-day PPA at 26 weeks, and no more than 5 cigarettes during the intervening period. Biochemically confirmed continuous abstinence was assessed at 26 weeks and was defined as self-reported continuous abstinence with a breath CO sample <10 ppm at both the associated 12- and 26-week biovalidation visits. Abstinence from all tobacco products was self-reported. NRT use included whether a participant ordered NRT (yes or no), and if so, what type of NRT they ordered, using participant-placed orders.

#### Participant Feedback

Participant feedback was sought on the setup, user experience, design, and impact of their assigned smoking cessation program. This included user satisfaction with the smoking cessation program (getting started with the program, program design, program was useful for quitting, program helped me quit, and program helped me stay quit). User satisfaction was also assessed through net promoter score (NPS), which queries the likelihood of recommending one’s program to a friend or colleague (scale 1-10) [43]. NPS is an industry indicator of participant loyalty to a product or service. NPS was calculated by subtracting the percentage of respondents who answered ≤6 (detractors) from the percentage of respondents who answered 9 or 10 (promotors).

#### Sample Size

As this is a pilot RCT and the first assessment of Pivot compared with usual care, the sample size is powered to show differences in engagement—specifically, the number of times participants opened their assigned app over the first 12 weeks of the study. In previous clinical studies, Pivot mean app openings were 24.2 to 38.7 (SD 20.8-25.9) by 90 days (data on file). In addition, Bricker et al [5] reported app openings comparing ACT-based smoking cessation apps (SmartQuit and iCanQuit) with QuitGuide. In a study by Bricker et al. [5], at 2-month follow-up, the authors reported that the mean app openings were 37.2 (SD 46.1) for SmartQuit and 15.2 (SD 13.6) for QuitGuide. In a subsequent study, at 12-month follow-up, the mean app openings were 37.5 (SD 88.4) for iCanQuit and 9.9 (SD 50.0) for QuitGuide [4].

On the basis of these data, we estimated a mean of 25 (SD 25) app openings in the Pivot intervention arm versus 15 (SD 19) app openings in the QuitGuide control arm at 12 weeks. Detecting a difference of 10 app openings between Pivot and QuitGuide with a power of 0.8 and an α of.05 would require 156.7 participants, which we round up to 158. In a previous study, 85.3% (272/319) of participants completed the end-of-Pivot questionnaire at a mean of 4.1 (SD 1.4) months after enrollment [44]. In assessing the primary end point at 3 months (12 weeks), we included an expected 15% attrition rate, with the aim to enroll up to 180 participants (up to 90 in each arm).

### Statistical Analyses

In this pilot RCT, differences between the Pivot intervention arm and the QuitGuide control arm were evaluated. Baseline comparisons and changes from baseline used unadjusted statistical tests. For numerical data, we calculated the mean (SD) and used a 2-tailed *t* test. For categorical data, we calculated the proportions and used the chi-square test or Fisher exact test. For results where a change from baseline can be measured, each participant’s baseline data served as their control to calculate a difference with a later time point (eg, CPD, SASEQ, STQ, and DTQ), which then served as the measurement, and a paired 2-tailed *t* test was used to test for a difference from 0.

For outcomes, regression analyses were adjusted for the randomization stratification covariates to detect differences between the treatment and control arms. Linear regression was used for numerical data to obtain a point estimate of the mean difference. For count outcomes, the incidence rate ratio (IRR) was estimated using Poisson regression when the variance to mean ratio was close to 1 or using negative binomial regressions when the variance to mean ratio was >1. For binary outcomes, the OR was estimated using logistic regression, and the relative risk was estimated using either log-link binomial regression or log-link Poisson regression with robust estimators [45]. For binary outcomes where there was a very high frequency response (eg, ≥95%), only the relative risk was presented. For multicategory outcomes of ≥3, multinomial logistic regression was used to test for proportion differences between the arms. If the multinomial logistic regression model did not converge, categories were collapsed. Statistical significance was set at *P*<.05. Analyses were conducted using SAS (version 9.4; SAS Institute).

In the assessment of quit rates (self-reported and biovalidated PPA and continuous abstinence and self-reported abstinence from all tobacco products), 2 sets of analyses were performed. In the ITT analysis, individuals who did not respond to PPA questions were assumed to be smoking. A study responder analysis was also performed, which only included individuals who completed the questionnaire from the associated time point. For the outcomes of quit attempts and the proportion who reduced CPD by at least 50%, a study completer analysis was performed.

### Data Collection

Data collection was performed via web-based questionnaires at baseline, weekly for the first 12 weeks, and at the 26-week follow-up. Collection of participant feedback on one’s assigned smoking cessation program was primarily over the first 12 weeks of the study to obtain input temporally closest to program use. Study data were imported directly into a secure database (PostgreSQL; PostgreSQL Global Development Group).

Participants were compensated for completing the web-based questionnaires, earning between US $10 and US $50 per questionnaire for up to US $265 in total for 14 questionnaires over the 26-week study period. Participants were compensated US $50 for each biovalidation visit they completed (up to 2 visits) for up to US $100. In total, participants could earn up to US $365 over the course of the 26-week study. Compensation was in the form of Visa or Mastercard gift cards that were mailed or emailed to their provided address approximately 2 to 3 weeks after completing the associated questionnaire(s) or biovalidation visits. Payments were bundled with participants receiving up to 4 payments over the 26-week course of the study. Remuneration was not tied to quitting smoking.

### Handling of Missing Data

Survey completion was high at 12 weeks, 97% (91/94) in QuitGuide and 98% (92/94) in Pivot, and at 6 months, 96% (90/94) in both QuitGuide and 96% (90/94) in Pivot. Therefore, completer and ITT analyses were considered appropriate at the 12- and 26-week time points.

The primary end point of the total number of app openings through 12 weeks was calculated by summing the number of weekly app openings, which were reported by the participants weekly and represented total app openings over the preceding 7 days. There were 170 participants who completed all 12 surveys. There were 4 participants (2 in Pivot and 2 in QuitGuide) who withdrew consent by week 3, accounting for 41 incomplete surveys. App openings for these participants were set to 0, as they did not participate in the study. This left 14 participants (8 Pivot and 6 QuitGuide) with one or more surveys not completed for 44 incomplete surveys. Although this only represented 7.4% of total participants and 2% of total surveys, imputation was necessary to calculate the total app openings, total days with app openings, and total weeks with app openings.

There was no pattern of missingness upon visual inspection, and multiple imputation method was performed using SAS multiple imputation procedure full conditional specification predicted mean matching with 25 imputations [46]. The primary end point of total app openings by the intervention and control arms was then compared in a negative binomial regression model adjusted for the 4 randomization covariates in each of the imputations with SAS MIANALYZE. Similarly, total days with app openings and total weeks with app openings were analyzed using negative binomial regression and Poisson regression, respectively. The mean of the imputed data was used for reporting descriptive statistics.

## Results

### Enrollment and Questionnaire Completion

From June 2021 to October 2021, a total of 3042 web-based screening forms were received; 533 (17.5%) met the screening eligibility criteria and responded to an initial outbound phone call from study staff. Of the 3042 individuals, 292 (9.6%) did not proceed further, most commonly (134/3042, 4.4%) owing to ineligibility after the phone call or lack of response to subsequent outreach (111/3042, 3.6%) after initial contact. Of 3042 individuals, 188 (6.2%) were randomized and completed enrollment (94 in each arm), comprising the ITT sample. All nonproportional quota sampling targets were achieved.

Because of the multistep enrollment process, the study slightly overenrolled by 4.4% (8/180 participants; 4 in each arm). Considering the minimal risk profile of the app-based smoking cessation programs and the ambulatory nature of the study in which participants completed the web-based questionnaires at their discretion, this overenrollment was not felt to be significant.

**Figure 1:**
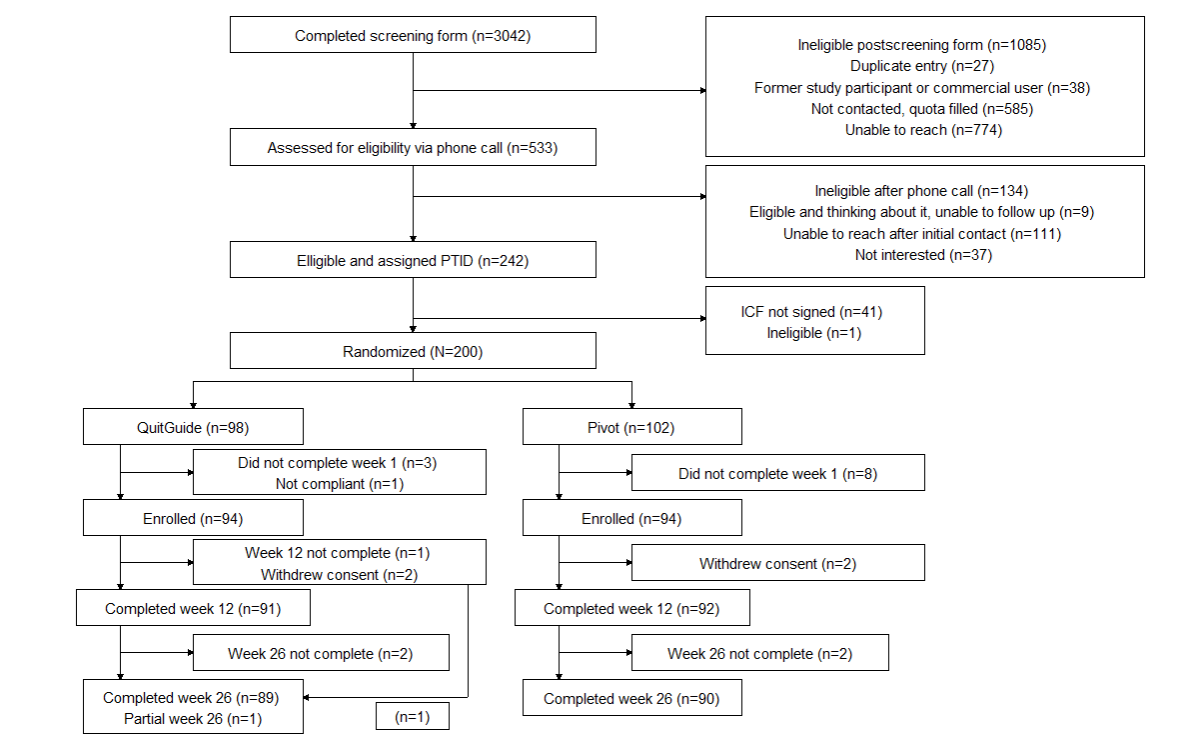
Study participant CONSORT (Consolidated Standards of Reporting Trials) flow diagram. ICF: informed consent form; PTID: participant identification number.

Study questionnaire completion rate was high; 97.3% (183/188) and 95.2% (179/188) of the participants completed the 12- and 26-week questionnaires, respectively, and comprised the study responder samples at those time points. A participant partially completed the 26-week questionnaire; in the associated study responder analyses, the denominator was 180. In each arm, 2 participants withdrew consent. Questionnaire completion rates did not differ between the 2 study arms. Study enrollment and attrition are depicted in the CONSORT (Consolidated Standards of Reporting Trials) flow diagram (Figure 1).

### Baseline Characteristics

The study sample had a mean age of 46.4 (SD 9.2) years, comprised 55.3% (104/188) women, was predominantly White (128/188, 68.1%), smoked a mean of 17.6 (SD 9.0) CPD at baseline, and had been smoking for a mean of 26.8 (SD 10.3) years. The mean Heaviness of Smoking Index was 3.2 (SD 1.2). Participants represented 42 of the 50 states in the United States along with the District of Columbia. The following states were not represented: Alaska, Delaware, Maine, Montana, North Dakota, New Hampshire, Vermont, and Wyoming. On average, participants had made 2.0 (SD 3.6) quit attempts over the past 12 months. Baseline demographic characteristics and smoking behavior were balanced between treatment groups at baseline. Participant baseline data are detailed in Table 1.

**Table 1:** Participant baseline data.

Characteristics				All (N=188)		Pivot (n=94)		QuitGuide (n=94)		*P* value	
**Demographics**											
	Age (years), mean (SD)			46.4 (9.2)		46.6 (10.1)		46.1 (8.2)		.70	
	Gender (women), n (%)			104 (55.3)		50 (53.2)		54 (57.5)		.56	
	**Ethnicity and race, n (%)**										.58
		White	128 (68.1)		66 (70.2)		62 (66)				
		Black	36 (19.2)		15 (16)		21 (22.3)				
		American Indian	1 (0.5)		1 (1.1)		0 (0)				
		Hispanic, Latino, or Spanish origin	13 (6.9)		8 (8.5)		5 (5.3)				
		Asian	1 (0.5)		0 (0)		1 (1.1)				
		Native Hawaiian	2 (1.1)		0 (0)		2 (2.1)				
		Some other race	3 (1.6)		2 (2.1)		1 (1.1)				
		Prefer not to answer	4 (2.1)		2 (2.1)		2 (2.1)				
	**Education, n (%)**										.63
		Less than 8th grade	1 (0.5)		0 (0)		1 (1.1)				
		Some high school	2 (1.1)		1 (1.1)		1 (1.1)				
		High school or General Educational Development	27 (14.4)		15 (16)		12 (12.8)				
		Some college	80 (42.6)		35 (37.2)		45 (47.9)				
		Associate’s (2 years) degree	28 (14.9)		13 (13.8)		15 (16)				
		Bachelor’s (4 years) degree	31 (16.5)		18 (19.2)		13 (13.8)				
		Master’s degree	15 (8)		10 (10.6)		5 (5.3)				
		Professional or doctorate degree	4 (2.1)		2 (2.1)		2 (2.1)				
	**Income (US $), n (%)**										.36
		<25,000	32 (17)		14 (14.9)		18 (19.2)				
		25,000-34,999	26 (13.8)		14 (14.9)		12 (12.8)				
		35,000-49,999	42 (22.3)		19 (20.2)		23 (24.5)				
		50,000-74,999	32 (17)		13 (13.8)		19 (20.2)				
		75,000-99,999	23 (12.2)		12 (12.8)		11 (11.7)				
		100,000-149,999	15 (8)		8 (8.5)		7 (7.5)				
		≥150,000	10 (5.3)		8 (8.5)		2 (2.1)				
		Prefer not to answer	8 (4.3)		6 (6.4)		2 (2.1)				
	**Employment, n (%)**										.83
		Yes, ≥20 hours per week	117 (62.2)		59 (62.8)		58 (61.7)				
		Yes, <20 hours per week	37 (19.7)		17 (18.1)		20 (21.3)				
		No	34 (18.1)		18 (19.2)		16 (17)				
	**Self-reported health, n (%)**										.34
		Excellent	5 (2.7)		4 (4.3)		1 (1.1)				
		Very good	57 (30.3)		24 (25.5)		33 (35.1)				
		Good	99 (52.7)		51 (54.3)		48 (51.1)				
		Fair	26 (13.8)		14 (14.9)		12 (12.8)				
		Poor	1 (0.5)		1 (1.1)		0 (0)				
	**Smartphone, n (%)**										.30
		iPhone	113 (60.1)		60 (63.8)		53 (56.4)				
		Android	75 (39.9)		34 (36.2)		41 (43.6)				
**Smoking and quitting behavior**											
	Cigarettes smoked per day, mean (SD)			17.6 (9)		18.0 (9.6)		17.2 (8.5)		.55	
	Years smoking, mean (SD)			26.8 (10.3)		27.7 (10.4)		25.8 (10.1)		.21	
	**First cigarette smoked after waking, n (%)**										.54
		Within 5 minutes	67 (35.6)		30 (31.9)		37 (39.4)				
		6-30 minutes	92 (48.9)		47 (50)		45 (47.9)				
		31-60 minutes	22 (11.7)		12 (12.8)		10 (10.6)				
		After 60 minutes	7 (3.7)		5 (5.3)		2 (2.1)				
	**Tobacco products used, n (%)**										.42
		Cigarettes only	162 (86.2)		79 (84)		83 (88.3)				
		Cigarettes+e-cigarettes or vaping	15 (8)		10 (10.6)		5 (5.3)				
		Cigarettes+cigars	3 (1.6)		1 (1.1)		2 (2.1)				
		Cigarettes+e-cigarettes or vaping+cigars	2 (1.1)		1 (1.1)		1 (1.1)				
		Cigarettes+chew or snuff	2 (1.1)		2 (2.1)		0 (0)				
		Cigarettes+e-cigarettes, vaping+chew, or snuff	1 (0.5)		0 (0)		1 (1.1)				
		Cigarettes+e-cigarettes or vaping+pipe	1 (0.5)		0 (0)		1 (1.1)				
		Cigarettes+hookah+cigars	1 (0.5)		0 (0)		1 (1.1)				
		Cigarettes+hookah	1 (0.5)		1 (1.1)		0 (0)				
	HSI^a^, mean (SD)			3.2 (1.2)		3.2 (1.3)		3.2 (1.2)		.72	
	Quit attempts in the past 12 months, mean (SD)			2.0 (3.6)		1.9 (3.4)		2.2 (3.8)		.63	
	**Methods used in past quit attempts^b^, n (%)**										
		Cold turkey	140 (74.5)		67 (71.3)		73 (77.7)		.72		
		NRT^c^	92 (48.9)		53 (56.4)		39 (41.5)		.06		
		e-Cigarettes or vaping	65 (34.6)		33 (35.1)		32 (34.0)		>.99		
		Varenicline or Chantix; Bupropion or Zyban or Wellbutrin	71 (37.8)		39 (41.5)		32 (34)		.37		
		None	16 (8.5)		5 (5.3)		11 (11.7)		.19		
		Hypnotherapy	11 (5.9)		7 (7.5)		4 (4.3)		.53		
		Quit Smoking classes	10 (5.3)		7 (7.5)		3 (3.2)		.33		
		Acupuncture	10 (5.3)		7 (7.5)		3 (3.2)		.33		
		Smartphone app	9 (4.8)		6 (6.4)		3 (3.2)		.49		
		Counseling	4 (2.1)		2 (2.1)		2 (2.1)		>.99		
		Other	4 (2.1)		1 (1.1)		3 (3.2)		.62		
	**Attitudes toward quitting smoking**										
		DTQ^d^, mean (SD)	3.5 (2.5)		3.5 (2.3)		3.6 (2.6)		.72		
		STQ^e^, mean (SD)	4.5 (2.4)		4.6 (2.4)		4.3 (2.3)		.33		
		SASEQ^f^, mean (SD)	11.7 (4.8)		11.8 (4.7)		11.5 (4.9)		.69		

^a^HSI: Heaviness of Smoking Index—low (0-1), medium (2-4), and high (5,6).

^b^Participants were asked to select all that apply.

^c^NRT: nicotine replacement therapy.

^d^DTQ: difficulty to stay quit—If you were to quit smoking right now, how difficult do you think it would be to stay smoke free? (1=really hard to stay quit; 10=really easy to stay quit).

^e^STQ: success to quit—If you were to quit smoking right now, how successful would you be? (1=not at all successful; 10=completely successful).

^f^SASEQ: Smoking Abstinence Self-efficacy Questionnaire (score 1-24).

### User Engagement and Retention

For the primary study outcome, Pivot participants self-reported a mean of 157.9 (SD 210.6) total app openings versus 86.5 (SD 66.3) in QuitGuide (IRR 1.8, 95% CI 1.4-2.3; *P*<.001) over the first 12 weeks of the study. The number of days with ≥1 app opening through 12 weeks was not different between the 2 groups: 49.6 (SD 24.1) in Pivot versus 50.4 (SD 25.2) in QuitGuide (IRR 1.0, 95% CI 0.8-1.1; *P*=.73). Also, the number of weeks with ≥1 app opening was not different between the 2 groups: 11.0 (SD 2.2) in Pivot versus 11.0 (SD 2.3) in QuitGuide (IRR 1.0, 95% CI 0.9-1.1; *P*=.91).

Self-report of logging into their app at least once a week was reported in ≥85% of the participants in each arm for each week through 12 weeks; in QuitGuide, it ranged from 85% to 97%, and in Pivot, it was 86% to 98%.

### Attitudes Toward Quitting Smoking

At 4 weeks, all responding participants indicated an ongoing desire to quit smoking (91/91, 100% in Pivot and 88/88, 100% in QuitGuide). Self-efficacy via SASEQ, STQ, and DTQ significantly increased in both groups from baseline to 12 weeks. The difference in these measures between the 2 groups at 12 weeks was not significant (Table 2).

**Table 2:** Changes in attitudes toward quitting smoking from baseline to 12 weeks (n=188).

Measure		All			QuitGuide			Pivot			*P* value^a^		Point estimate^b^ (95% CI)		*P* value
		Value, n (%)	Value, mean (SD)	Value, n (%)		Value, mean (SD)	Value, n (%)		Value, mean (SD)						
**SASEQ^c^**															
	Baseline	188 (100)	11.7 (4.8)	94 (100)		11.5 (4.9)	94 (100)		11.8 (4.7)	.69		—^d^		—	
	12 weeks	183 (97.3)	14.3 (6.5)	91 (97)		14.1 (6.2)	92 (98)		14.5 (6.9)	—		0.32 (−1.5 to 2.2)		.73	
	Change	—	2.7 (7.3)	—		2.7 (7.9)^e^	—		2.6 (6.7)^f^	—		0.08 (−2.0 to 2.2)		.94	
**STQ^g^**															
	Baseline	188 (100)	4.5 (2.4)	94 (100)		4.3 (2.3)	94 (100)		4.6 (2.4)	.33		—		—	
	12 weeks	183 (97.3)	6.2 (3.1)	91 (97)		5.8 (3.1)	92 (98)		6.6 (3.0)	—		0.79 (−0.08 to 1.6)		.07	
	Change	—	1.8 (3.6)	—		1.6 (3.6)^e^	—		2.0 (3.6)^e^	—		0.41 (−0.57 to 1.4)		.41	
**DTQ^h^**															
	Baseline	188 (100)	3.5 (2.5)	94 (100)		3.6 (2.6)	94 (100)		3.5 (2.3)	.72		—		—	
	12 weeks	183 (97.3)	5.4 (3.1)	91 (97)		5.2 (3.0)	92 (98)		5.7 (3.1)	—		0.47 (−0.40 to 1.3)		.29	
	Change	—	1.9 (3.5)	—		1.7 (3.4)^e^	—		2.2 (3.7)^e^	—		0.59 (−0.29 to 1.5)		.18	

^a^2-tailed *t* test.

^b^Point estimate obtained from linear regression adjusted with randomization covariates: daily smoking frequency (≤14 vs ≥15 cigarettes per day), employment status (full-time or part-time employment vs not employed), race and ethnicity (minority race and ethnicity vs non-Hispanic White) and expected difficulty staying quit (scale 1-10; self-reported score of ≤5 vs ≥6).

^c^SASEQ: Smoking Abstinence and Self-efficacy Questionnaire (score 1-24).

^d^Not available.

^e^Paired *t* test difference from baseline to 12 weeks; *P*<.001.

^f^Paired *t* test difference from baseline to 12 weeks; *P*=.001.

^g^STQ: success to quit—If you were smoking right now, how successful would you be? (1=not at all successful; 10=completely successful).

^h^DTQ: difficulty to stay quit—If you were to quit smoking right now, how difficult do you think it would be to stay smoke free? (1=really hard to stay quit; 10=really easy to stay quit).

### Smoking Behavior

#### Quit Attempts

Overall, 96.6% (173/179) of the responders reported making at least 1 quit attempt through 26 weeks, with comparable proportions in each study group: Pivot, 96% (86/90) and QuitGuide, 98% (87/89; RR Poisson 1.0, 95% CI 0.9-1.0; *P*=.41). On average, QuitGuide participants reported more quit attempts: Pivot, 4.2 (SD 4.4) versus QuitGuide, 6.3 (SD 6.1; IRR negative binomial 0.7, 95% CI 0.5-0.9; *P*=.003).

#### Change in CPD

Among participants who responded at 26 weeks (N=180), CPD were reduced by 62.6% (SD 38.1%) from baseline. Within each group, the reduction in CPD from baseline to 26 weeks was significant (*P*<.001 for both). The CPD reduction was similar between the 2 groups: Pivot, −62.1% (SD 40.3%) versus QuitGuide, −63.1% (SD 35.9%; point estimate 1.1, 95% CI −9.9 to 12.0; *P*=.85).

Among participants who did not report 7-day (or greater) PPA at 26 weeks (N=121), CPD were reduced by 44.4% (SD 33.7%) from baseline. Within each group, the reduction in CPD from baseline to 26 weeks was significant (*P*<.001 for both). The reduction in CPD was similar between the 2 groups: Pivot, −39.1% (SD 34.7%) versus QuitGuide, −48.9% (SD 32.5%; point estimate 11.6, 95% CI −0.4 to 23.6; *P*=.06).

Among participants who responded at 26 weeks (N=180), the proportion who reduced CPD by ≥50% was similar between the 2 groups: Pivot, 62% (56/90) versus QuitGuide, 66% (59/90; OR 0.9, 95% CI 0.5-1.7; *P*=.65; RR 1.0, 95% CI 0.8-1.2; *P*=.77).

Focusing on participants who did not report 7-day (or greater) PPA at 26 weeks (N=121), the proportion who reduced CPD by ≥50% was similar between the 2 groups: Pivot, 39% (22/56) versus QuitGuide, 52% (34/65; OR 0.5, 95% CI 0.2-1.1; *P*=.10; RR 0.7, 95% CI 0.5-1.1; *P*=.09).

#### Cessation Rates

Cessation rates included self-reported 7- and 30-day PPA, continuous abstinence and abstinence from all tobacco products, and biochemically confirmed abstinence and biochemically confirmed continuous abstinence, as detailed in Table 3. At 12 and 26 weeks, differences between the 2 study groups in self-reported 7- and 30-day PPA rates and abstinence from all tobacco products were not statistically significant. By contrast, differences in biochemically confirmed abstinence and biochemically confirmed continuous abstinence rates were significant at 12 and 26 weeks. At 12 weeks, biochemically confirmed abstinence (ITT) was achieved in 29% (27/94) of the Pivot participants versus 13% (12/94) of the QuitGuide participants (OR 2.8, 95% CI 1.3-6.1; *P*=.008; RR 2.3, 95% CI 1.2-4.2; *P*=.008). At 26 weeks, biochemically confirmed continuous abstinence (ITT) was achieved in 21% (20/94) of the Pivot participants versus 10% (9/94) of the QuitGuide participants (OR 2.7, 95% CI 1.1-6.4; *P*=.03; RR 2.2, 95% CI 1.1-4.6; *P*=.03). Notably, at 12 and 26 weeks, the participation rate in biovalidation visits was high (84.7% overall; >80% for each group at 12 and 26 weeks) and was comparable between the 2 groups.

**Table 3:** Smoking cessation rates at 12 and 26 weeks (n=188).

Outcome		Overall, n (%)	Pivot, n (%)	QuitGuide, n (%)	Odds ratio (95% CI)	*P* value	Relative risk (95% CI)^a^	*P* value
**12 weeks**								
	7-day PPA^b^; ITT^c^	59 (31.4)	33 (35.1)	26 (27.7)	1.4 (0.8-2.7)	.28	1.2 (0.8-1.8)	.50
	7-day PPA; responder^d^	59 (32.2)	33 (35.9)	26 (28.6)	1.4 (0.8-2.7)	.30	1.2 (0.8-1.8)	.53
	30-day PPA; ITT	48 (25.5)	27 (28.7)	21 (22.3)	1.4 (0.7-2.8)	.32	1.2 (0.7-1.9)	.56
	30-day PPA; responder^d^	48 (26.2)	27 (29.3)	21 (23.1)	1.4 (0.7-2.7)	.35	1.2 (0.7-1.9)	.59
	Biochemically confirmed abstinence; ITT^e^	39 (20.7)	27 (28.7)	12 (12.8)	2.8 (1.3-6.1)	.008	2.3 (1.2-4.2)	.008
	Biochemically confirmed abstinence; responder^d,e^	39 (21.3)	27 (29.3)	12 (13.2)	2.8 (1.3-6.1)	.009	2.3 (1.2-4.1)	.009
	Self-reported abstinence from all tobacco products; ITT	56 (29.8)	31 (33)	25 (26.6)	1.2 (0.6-2.3)	.56	1.1 (0.7-1.6)	.82
	Self-reported abstinence from all tobacco products; responder^d^	56 (30.6)	31 (33.7)	25 (27.5)	1.2 (0.6-2.2)	.60	1.0 (0.7-1.6)	.87
**26 weeks**								
	7-day PPA; ITT^c^	59 (31.4)	34 (36.2)	25 (26.6)	1.7 (0.9-3.2)	.12	1.3 (0.8-1.9)^f^	.27
	7-day PPA; responder^g^	59 (32.8)	34 (37.8)	25 (27.8)	1.7 (0.9-3.2)	.13	1.5 (1.0-2.3)	.06
	30-day PPA; ITT	51 (27.1)	30 (31.9)	21 (22.3)	1.7 (0.9-3.4)	.12	1.4 (0.9-2.2)	.18
	30-day PPA; responder^g^	51 (28.3)	30 (33.3)	21 (23.3)	1.7 (0.9-3.4)	.13	1.4 (0.9-2.22)	.19
	Biochemically confirmed abstinence; ITT^h^	40 (21.3)	26 (27.7)	14 (14.9)	2.3 (1.1-4.8)	.03	1.9 (1.1-3.5)	.02
	Biochemically confirmed abstinence; responder^g,h^	40 (22.2)	26 (28.9)	14 (15.6)	2.3 (1.1-4.8)	.03	1.9 (1.1-3.4)	.02
	Self-reported continuous abstinence; ITT	39 (20.7)	24 (25.5)	15 (16.0)	1.9 (0.9-3.8)	.10	1.6 (0.9-2.8)	.11
	Self-reported continuous abstinence; responder^i^	39 (21.8)	24 (26.7)	15 (16.9)	1.8 (0.9-3.9)	.11	1.6 (0.9-2.8)	.12
	Biochemically confirmed continuous abstinence; ITT	29 (15.4)	20 (21.3)	9 (9.6)	2.7 (1.1-6.4)	.03	2.2 (1.1-4.6)^f^	.03
	Biochemically confirmed continuous abstinence; responder^i^	29 (16.2)	20 (22.2)	9 (10.1)	2.7 (1.1-6.3)	.03	2.3 (1.1-4.7)	.02
	Self-reported abstinence from all tobacco products; ITT	55 (29.3)	32 (34)	23 (24.5)	1.6 (0.9-3.1)	.13	1.5 (1.0-2.3)	.06
	Self-reported abstinence from all tobacco products; responder^g^	55 (30.6)	32 (35.6)	23 (25.6)	1.6 (0.8-3.1)	.16	1.5 (1.0-2.2)	.08

^a^Negative binomial regression.

^b^PPA: point prevalence abstinence.

^c^ITT: intention to treat; total N=188: 94 in Pivot and 94 in QuitGuide.

^d^Responders to 12-week questionnaire; N=183 total: 92 in Pivot and 91 in QuitGuide.

^e^Completers of 12-week biovalidation visit; n=50 total: 29 in Pivot and 21 in QuitGuide.

^f^Log-link Poisson regression.

^g^Responders to 26-week questionnaire; N=180 total: 90 in Pivot and 90 in QuitGuide (includes responses from 1 partial responder).

^h^Completers of 26-week biovalidation visit; n=50 total: 29 in Pivot and 21 in QuitGuide.

^i^Responders to 26-week questionnaire; N=179 total: 90 in Pivot and 89 in QuitGuide.

#### Use of NRT

At 26 weeks, 99% (93/94) of the Pivot participants had ordered NRT compared with 82% (77/94) of the QuitGuide participants (RR 1.2, 95% CI 1.1-1.3; *P*<.001). The average number of NRT orders placed per participant was 3.1 (SD 1.9) in Pivot and 1.6 (SD 1.5) in QuitGuide (IRR 1.9, 95% CI 1.5-2.3; *P*<.001). Combination therapy (patch+gum or patch+lozenge) was the most common regimen among participants (Table 4).

**Table 4:** Nicotine replacement therapy (NRT) orders placed by participants through 26 weeks (*P*<.001)^a^.

	All (n=188), n (%)	Pivot (n=94), n (%)	QuitGuide (n=94), n (%)
≥1 NRT single therapy^b^ order	31 (16.5)	23 (24.5)	8 (8.5)
≥1 NRT combination therapy^c^ order	101 (53.7)	44 (46.8)	57 (60.6)
≥1 NRT single therapy order+≥1 NRT combination therapy order	38 (20.2)	26 (27.7)	12 (12.8)
None	18 (9.6)	1 (1.1)	17 (18.1)
Total	188 (100)	94 (100)	94 (100)

^b^Single therapy: nicotine patch alone, nicotine gum alone, or nicotine lozenge alone.

^c^Combination therapy: nicotine patch+nicotine gum or nicotine patch+nicotine lozenge.

^a^Multinomial logistic regression adjusted for randomization covariates.

#### Participant Feedback

In general, participant feedback was more favorable for the Pivot program (Multimedia Appendix 1). The Pivot program was ranked as easier to set up and start using (scale 1-10, higher value equates to easier): Pivot, 8.2 (SD 2.3) versus QuitGuide, 7.1 (SD 3.0; point estimate 1.0, 95% CI 0.2-1.8; *P*=.01). In both groups, high proportions of participants indicated their study program helped them with their goals related to smoking (true or false): Pivot, 86% (79/92) versus QuitGuide, 76% (69/91; OR 2.0, 95% CI 0.9-4.2; *P*=.08; RR 1.1, 95% CI 1.0-1.3; *P*=.17). Among participants who reported 7-day PPA at 6 months (n=59), most reported their study program helped them quit smoking (true or false): Pivot, 100% (34/34) versus QuitGuide, 88% (22/25; RR 1.1, 95% CI 1.0-1.3; *P*=.08).

NPS was sought at 4, 12, and 26 weeks and was significantly higher for Pivot at each time point (Multimedia Appendix 2). Specifically, at 4, 12, and 26 weeks, the NPS for Pivot versus QuitGuide was 50.6 versus 1.1, 44.6 versus 11.0, and 57.8 versus 23.6, respectively.

## Discussion

### Principal Findings

This pilot RCT compared user engagement and retention, change in attitudes toward quitting smoking, change in smoking behavior, and participant feedback in adult smokers randomized to either the Pivot or QuitGuide app-based smoking cessation programs. Program engagement as assessed by total app openings through 12 weeks, the preregistered primary outcome of the study, was significantly higher in Pivot than in QuitGuide (*P*<.001). Measures assessing attitudes toward quitting smoking, including SASEQ, STQ, and DTQ, improved significantly in each group through 12 weeks but were not different among groups. Most (173/188, 92%) participants made at least 1 quit attempt, with QuitGuide participants reporting more quit attempts through 26 weeks (*P*=.003). The study was not powered for differences in quit rates; although self-reported 7- and 30-day quit rates were approximately 10 percentage points higher in Pivot at 26 weeks (eg, 7-day PPA at 26 weeks was 36.2% in Pivot and 26.6% in QuitGuide; ITT), these differences were not statistically significant. However, differences in biovalidated quit rates were significant at 12 weeks (28.7% Pivot vs 12.8% QuitGuide, ITT; *P*=.008) and 26 weeks (27.7% Pivot vs 14.9% QuitGuide, ITT; *P*=.03), as was the difference in the biovalidated continuous quit rate at 26 weeks (21.3% Pivot vs 9.6% QuitGuide, ITT; *P*=.03). In general, participants rated the Pivot program more favorably, including the setup and impact of the program and the likelihood of recommending their program to a friend or colleague.

### Comparison With Prior Work

#### Engagement

For self-reported app openings, the primary outcome of user engagement, both study arms had greater engagement than expected. At 12 weeks, Pivot had an average of 157.9 total app openings and QuitGuide had 86.5, greater than the self-reported 37.2 average app openings for the SmartQuit arm and 15.2 for the QuitGuide arm reported at 8 weeks in the study by Bricker et al [5]. Although both studies used weekly or biweekly email engagement reminders, this study collected use data through weekly web-based questionnaires for 12 weeks, whereas Bricker et al [5] collected use data at 2 months after randomization. In addition, these data represent 1 more month of app use (12 weeks) than those reported by Bricker et al [5] (8 weeks). These study design differences could have contributed to the increase in self-reported app use between the 2 studies. For additional context, the following number of app openings for digital smoking cessation programs were reported through in-app data or Google Analytics (not self-report) in other studies: mean 100.6 app openings at 8 weeks with Clickotine [47], mean 37.5 app openings at 12 months with iCanQuit [4], and mean 37 app openings at 4 weeks after quit date with Quit Genius [38].

#### Smoking Cessation

Digital smoking cessation interventions have a wide range in their offerings. In a broad assessment of digital smoking cessation interventions with outcomes at 6 months, 7-day PPA rates range from 9.8% to 33.9% [4,48-51]. In this study, 36% (34/94) of the Pivot participants reported 7-day PPA, which was slightly above this range. This higher quit rate may reflect the multifaceted nature of Pivot—smartphone app, coaching, medication, and personal CO breath sensor—yielding a variety of smoking cessation tools to support its users.

Narrowing the scope to studies with smoking cessation programs similar to Pivot, 7-day PPA outcomes at 6 months range from 33.9% to 35.9% [4,7,16]; Pivot’s 7-day PPA rate of 36.2% is comparable. Similarly, 32% (30/94) of the Pivot participants achieved 30-day PPA at 6 months, rates that are similar to the 25% to 31.3% previously reported [4,16]. Published continuous abstinence rates at 6 months from smoking cessation programs similar to Pivot range from 23.8% to 27.2% [7,16]; the rate of 25.5% from this study again aligns with these results.

Comparison of cessation outcomes with QuitGuide is limited; however, Bricker et al [4] reported that 24% and 14.7% of participants achieved 7- and 30-day PPA at 6 months [4], respectively, compared with 26.6% and 22.3% in this study.

Making direct comparisons between Pivot and similar programs is somewhat limited owing to differences in study design, data collection time points, and study populations but does provide context to consider these results. For differences that are relevant for the aforementioned comparisons, examples include lack of NRT provision, CO breath sensor, coaching, and biovalidation in the studies by Bricker et al [4,5] and biovalidation of a minority of participants in the study by Webb et al [7,38]. Nonetheless, the growing body of outcome data for digital, app-based smoking cessation interventions, with similar quit rates from different investigator groups, increases confidence and credibility in this approach to cessation.

#### Self-reported Versus Biovalidated Abstinence Rates

Biovalidated abstinence rates and continuous abstinence rates were lower than the associated self-reported rates in both groups, although this was more pronounced in the QuitGuide arm. The first contributing factor was participants who did not schedule or did not attend their scheduled videoconference biovalidation visit. This accounted for 12% to 19% of potential eligible participants and was not different between the 2 study arms; similar and higher attrition rates have been reported elsewhere [7,38,52]. Notably, the discrepancy was primarily due to a change in smoking status (ie, relapse in the last 7 days since completing the associated study questionnaire) as reported at the outset of the biovalidation visits; this occurred in 15% (15/100) of all completed visits, specifically in 26% (11/42) of the 12- and 26-week visits completed by QuitGuide participants and in 10% (4/58) of these visits completed by Pivot participants. Obtaining a CO breath sample value that was discordant with self-reported abstinence was less common and occurred in 6% (6/100) of all completed visits, specifically in 12% (5/42) of the 12- and 26-week visits completed by QuitGuide participants and in 2% (1/58) of these visits completed by Pivot participants.

These results suggest a role of quit status instability in the discrepancy between self-reported and biovalidated quit rates in this study, particularly among QuitGuide participants. Although we believe it is less likely, we also cannot exclude the possibility of inaccurate self-reporting of quit status on the study questionnaires, leading to the scheduling of biovalidation visits; the expected effect would be inflated self-reported quit rates. Inaccurate self-reporting could be the result of motivation to seek additional compensation through the biovalidation visits or the result of differential experience between the 2 study arms with the breath sensor. Regarding compensation as a motivation, in the background of comparable socioeconomic characteristics in the 2 study arms, we have no reason to expect this to show up disproportionately in one arm over the other. Moreover, if compensation were a significant motivator, we might expect “repeat offenders”: participants who self-reported abstinence on their questionnaires, then declared relapse at the outset of the subsequent biovalidation visit at both 12 and 26 weeks. However, none of the participants demonstrated this behavior. The impact of previous experience with a CO breath sensor was explored in the RCT conducted by Webb et al [7,38], in 530 adult smokers in which CO breath sensors were provided to 50% of all participants in both arms. The investigators reported, “Whether or not a participant was provided with a CO device did not significantly predict quit rate (*P*=.29 in logistic regression with CO device and intervention main effects).”

Comparison to previous studies is challenging owing to differences in methodology. However, our results relating to the decrease from self-reported to CO-biovalidated quit rates seem to fall in the range of those previously reported. Webb et al [7,38] conducted an RCT of 530 adult smokers in the United Kingdom, randomized to an app-based clinician-assisted smoking cessation program (Quit Genius) or Very Brief Advice. They reported that breath sample results corresponded with self-reported abstinence in 93.6% of the participants at 26 weeks. Notably, biovalidation was performed in a minority (approximately 40%) of self-reported abstainers. Piper et al [52] conducted an RCT in 623 adult smokers in the United States randomized to recommended usual care (10 minutes of in-person counseling, 8 weeks of nicotine patch, and referral to quitline services) or abstinence-optimized treatment (3 weeks of prequit mini lozenges, 26 weeks of nicotine patch+mini lozenges, 3 in-person and 8 phone counseling sessions, and 7-11 automated calls to prompt medication use). In contrast to the study by Webb et al [7,38], Piper et al [52] reported biovalidation rates that were less than half of self-reported abstinence rates (eg, 39.3% self-reported 7-day PPA decreased to 15.9% biochemically confirmed abstinence in the abstinence-optimized treatment group at 26 weeks). Finally, Garrison et al [48] assessed CO-confirmed abstinence rates in an RCT assessing app-based mindfulness training with experience sampling versus experience sampling alone in 325 adult smokers. They reported an overall 18.2% self-reported 7-day PPA rate compared with 11.1% overall CO-verified abstinence rate at 6 months. Characteristics of study design and population have been shown to influence biovalidation rates. Some such relevant factors are present in this and aforementioned studies, including varying degrees of contact from minimal to face to face and varying cessation program intensity [52]. As the body of evidence on biovalidation continues to grow, so too will more informed narratives of optimal use and appropriate expectations for differences in self-reported versus biovalidated quit rates.

#### Notable Similarities and Differences Among Study Group Outcomes

Both groups had significant increases in measures of self-efficacy and confidence in quitting at 12 weeks, but these differences were not significant between the study groups. Both groups also reported significant decreases in CPD over time, with approximately 40% to 50% of those who did not achieve 7-day PPA in each group reducing their CPD by ≥50% at 26 weeks; again, these differences were not significant among the groups. In the setting of biovalidated quit rates that were significantly different among the study groups, it is interesting to note these milestones did not track in a similar fashion, considering they have historically been associated with an increased likelihood of quitting smoking [16,51,53-57].

The study groups did have significant differences in program engagement, with more program use, via total app openings, in the Pivot group. This finding aligns with the higher biovalidated abstinence and biovalidated continuous abstinence rates in the Pivot group. Study participants in both groups reported comparable number of days and duration (in weeks) of program use. Accordingly, the higher total app openings reported in Pivot suggests greater use per day. Higher program engagement has been associated with better outcomes in app-based smoking cessation programs [4,51,58]. Although the primary outcome sought to compare engagement of the holistic Pivot and QuitGuide smoking cessation programs via app openings, it is worth noting the possible influence on app use patterns of specific features in Pivot that are not present in QuitGuide, such as the breath sensor and in-app coach messaging. The intent and design of this study was not conducive to the assessment of individual app functions in facilitating engagement, which is a topic requiring finer discriminatory evaluation (such as A/B testing) that is of interest for future studies.

Another difference between the 2 study groups includes more quit attempts per person in QuitGuide. Coupled with the lower biovalidated abstinence and continuous abstinence rates in this group largely due to short-term relapse, this suggests less stability of quit among the QuitGuide users. Similar findings of higher quit attempts with lower quit rates have been reported in control arms elsewhere [7]. Finally, NRT use was higher in the Pivot study arm, which one would expect in this group with higher biovalidated abstinence and continuous abstinence rates. Although both study groups had access to free NRT with standardized repeated reminders of this access, it is likely that a more comprehensive incorporation of NRT in the Pivot program, through both education and support by tobacco cessation coaches contributed to the increased NRT use in this group.

### Strengths and Limitations

This study had several strengths. First, the study population was diverse and balanced, with nonproportional quota sampling goals achieved. Also, the comparison of same-modality interventions, with the control being a well-established and well-studied app-based cessation program helps minimize potential modality-related confounding and provides context for our results. Another strength is the inclusion of biovalidation for all those who reported 7-day or greater abstinence at 12 and 26 weeks. In addition, all the following metrics were robust: participant retention (approximately 98% in each arm), survey completion rates (≥92% for each survey), and biovalidation visit completion rates (approximately 85% overall).

This study also had several limitations. First, the inclusion criterion of intention to quit in the next 30 days resulted in a study population that may not reflect the general population of smokers. Aggregating across studies and populations, Prochaska et al [55] estimated that at any given time, approximately 20% of smokers were thinking of quitting smoking in the next 30 days, 35% to 40% were thinking of quitting in the next 6 months, and 40% to 45% were not seriously thinking of quitting. In a previous cohort study of Pivot in which the study population more closely aligned with the general population of smokers (66% were not seriously thinking of quitting in the next 30 days at study entry), this factor was not predictive of cessation outcomes. Furthermore, there is some benefit to this inclusion criterion in that this aspect of our study population matches similar previous studies, more readily facilitating comparison.

Second, the self-reporting of engagement, including app openings is not as accurate as the report of such data through in-app or Google Analytics use data. However, we did not have this capability with QuitGuide. Although we expect this might result in overestimation of app openings, we have no reason to believe participants in either arm would be more likely to do so. The fact that the study arms reported a similar number of days and weeks of app use lends further credibility to the reported differential in app openings.

Third, as a pilot RCT and the first comparison of Pivot to comparable usual care, this study was not powered for cessation outcomes. Differences in self-reported abstinence were not significant, whereas differences in biovalidated abstinence rates were. It is unclear whether a larger study would have yielded significant differences in self-reported cessation outcomes or biovalidated abstinence rates; that question remains to be answered in a study powered accordingly.

Fourth, the Pivot program included additional cessation tools that the QuitGuide program did not, including a CO breath sensor, access to SMS text messaging–based coaching with a tobacco cessation coach, and a moderated web-based community support forum. The study compares the holistic programs but is limited in that it cannot determine if, and to what extent, any of these features in Pivot were more effective than the QuitGuide app plus NRT.

Fifth, the possible impact of compensation must also be considered. We took steps to minimize the impact of compensation, including conservative payment amounts that were commensurate with participant effort, delaying payment by 2 to 3 weeks from completion of compensated events, and not tying compensation to outcomes. Nonetheless, we cannot exclude some influence of study payment on participant behavior.

Sixth, after randomization, all researchers were unblinded to participant group allocation. This can have implications for study conduct such as possible unbalanced participant communication and data collection efforts. Accordingly, we designed the study with mitigating factors such as scheduled, standardized, and scripted written and verbal participant communications that were reviewed by the institutional review board. We believe that the high and comparable questionnaire and biovalidation visit completion rates (>92% for questionnaires and >80% for biovalidation visits at 12 and 26 weeks in both study arms) reflect favorably on our attempt to minimize the possible effect of unblinded researchers.

Seventh, exhaled CO as a biovalidation test for smoking cessation is imperfect. The half-life of CO is, on average, 4 hours and is influenced by activity level (ie, shorter half-life when exercising and longer when sleeping). Accordingly, smokers may be able to abstain from smoking for several hours before providing a breath sample and obtain a CO value consistent with “not smoking”; we cannot exclude this occurrence in the biovalidation visits. Moreover, secondhand smoke, use of other combustible substances such as marijuana, and environmental or occupational CO exposure can increase CO levels. That said, the limitations of other biovalidation methods made exhaled CO, which is noninvasive, less expensive, and easy for a lay user to perform the preferred option. Specifically, although cotinine, a nicotine metabolite, has a longer half-life (≥8-30 hours) than CO and therefore requires longer abstinence periods (2-7 days) to reach “nonsmoking” levels, its collection from body fluids is more onerous and will yield positive results in individuals using NRT, which was problematic with our study design. Anabasine and anatabine are minor tobacco alkaloids that are specific for tobacco-derived products (eg, cigarettes, cigars, and smokeless tobacco). They are well suited for testing individuals for tobacco use who are using NRT. However, these biomarkers require urine collection and chromatography–mass spectrometry measurement [59]. Altogether, when considering the remote nature of this study and the provision of NRT, we felt exhaled CO, despite its imperfections, was our best option for biovalidation.

Finally, it should be noted that recruitment, enrollment, and study conduct were performed during the COVID-19 pandemic and during a time characterized by heightened social, political, and economic stressors. Although it is beyond the scope of the study to quantify these factors, it is worth noting as this is a difference between this study and the aforementioned comparator studies. The impact is unknown at this time.

### Conclusions

Previous cohort studies assessing Pivot established the foundation for further comparative assessment, leading to this study. This RCT compared Pivot with a well-established app-based smoking cessation program and found that Pivot produced higher engagement, higher biovalidated cessation rates, and more favorable user feedback. This study, with 6-month outcomes, supports the efficacy and durability of Pivot and adds to the growing body of evidence identifying an emerging role for digital, app-based interventions for smoking cessation. As the data narrative for rising digital smoking cessation programs unfolds, areas ripe for future assessment include longer-term durability data, evaluation of the contributions to program engagement and abstinence rates of individual app functions such as coaching and breath sensor result tracking, and assessment of the cost-effectiveness of digital app-based interventions.

## Appendix

Multimedia Appendix 1 Participant feedback.

Multimedia Appendix 2 Net promoter score.

Multimedia Appendix 3 CONSORT-eHEALTH checklist (V 1.6.1) [60].

## Abbreviations

ACT: acceptance and commitment therapy
CBT: cognitive behavioral therapy
CO: carbon monoxide
CONSORT: Consolidated Standards of Reporting Trials
CPD: cigarettes per day
DTQ: difficulty to stay quit
FDA: Food and Drug Administration
IRR: incidence rate ratio
ITT: intention to treat
NPS: net promoter score
NRT: nicotine replacement therapy
OR: odds ratio
PPA: point prevalence abstinence
ppm: parts per million
RCT: randomized controlled trial
RR: relative risk ratio
SASEQ: Smoking Abstinence Self-efficacy Questionnaire
STQ: success to quit
USCPG: US Clinical Practice Guideline

## Data Availability

Deidentified data will be shared on reasonable request, stating the purpose, to the corresponding author, which is subject to approval by the sponsor.

## References

[1] GBD 2019 Tobacco Collaborators (2021). Spatial, temporal, and demographic patterns in prevalence of smoking tobacco use and attributable disease burden in 204 countries and territories, 1990-2019: a systematic analysis from the Global Burden of Disease Study 2019. Lancet.

[2] Babb S, Malarcher A, Schauer G, Asman K, Jamal A (2017). Quitting smoking among adults - United States, 2000-2015. MMWR Morb Mortal Wkly Rep.

[3] Whittaker R, McRobbie H, Bullen C, Rodgers A, Gu Y, Dobson R (2019). Mobile phone text messaging and app-based interventions for smoking cessation. Cochrane Database Syst Rev.

[4] Bricker JB, Watson NL, Mull KE, Sullivan BM, Heffner JL (2020). Efficacy of smartphone applications for smoking cessation: a randomized clinical trial. JAMA Intern Med.

[5] Bricker JB, Mull KE, Kientz JA, Vilardaga R, Mercer LD, Akioka KJ, Heffner JL (2014). Randomized, controlled pilot trial of a smartphone app for smoking cessation using acceptance and commitment therapy. Drug Alcohol Depend.

[6] BinDhim NF, McGeechan K, Trevena L (2018). Smartphone Smoking Cessation Application (SSC App) trial: a multicountry double-blind automated randomised controlled trial of a smoking cessation decision-aid 'app'. BMJ Open.

[7] Webb J, Peerbux S, Ang A, Siddiqui S, Sherwani Y, Ahmed M, MacRae H, Puri H, Majeed A, Glasner S (2022). Long-term effectiveness of a clinician-assisted digital cognitive behavioral therapy intervention for smoking cessation: secondary outcomes from a randomized controlled trial. Nicotine Tob Res.

[8] Pechmann C, Delucchi K, Lakon CM, Prochaska JJ (2017). Randomised controlled trial evaluation of Tweet2Quit: a social network quit-smoking intervention. Tob Control.

[9] Smokefree. U.S. Department of Health and Human Services, National Institutes of Health, National Cancer Institute.

[10] Abrams DB, Niaura R, Brown RA, Emmons KM, Goldstein MG, Monti PM (2008). The Tobacco Dependence Treatment Handbook: A Guide to Best Practices.

[11] Beard E, West R (2012). Pilot study of the use of personal carbon monoxide monitoring to achieve radical smoking reduction. J Smok Cessat.

[12] Marler JD, Fujii CA, Wong KS, Galanko JA, Balbierz DJ, Utley DS (2020). Assessment of a personal interactive carbon monoxide breath sensor in people who smoke cigarettes: single-arm cohort study. J Med Internet Res.

[13] Graham AL, Papandonatos GD, Erar B, Stanton CA (2015). Use of an online smoking cessation community promotes abstinence: results of propensity score weighting. Health Psychol.

[14] Sadasivam RS, Allison JJ, Ray MN, Ford DE, Houston TK (2012). Using a resource effect study pre-pilot to inform a large randomized trial: the Decide2Quit.Org Web-assisted tobacco intervention. AMIA Annu Symp Proc.

[15] Sadasivam RS, Kamberi A, DeLaughter K, Phillips B, Williams JH, Cutrona SL, Ray MN, Gilbert GH, Houston TK, QUITPRIMO, National Dental PBRN Collaborative Group (2020). Secure asynchronous communication between smokers and tobacco treatment specialists: secondary analysis of a Web-assisted tobacco intervention in the QUIT-PRIMO and National Dental PBRN Networks. J Med Internet Res.

[16] Marler JD, Fujii CA, Galanko JA, Balbierz DJ, Utley DS (2021). Durability of abstinence after completing a comprehensive digital smoking cessation program incorporating a mobile app, breath sensor, and coaching: cohort study. J Med Internet Res.

[17] (2021). The Employment Situation - March 2021. Bureau of Labor Statistics, U.S. Department of Labor.

[18] Prochaska JJ, Michalek AK, Brown-Johnson C, Daza EJ, Baiocchi M, Anzai N, Rogers A, Grigg M, Chieng A (2016). Likelihood of unemployed smokers vs nonsmokers attaining reemployment in a one-year observational study. JAMA Intern Med.

[19] Fielding-Singh P, Vogel EA, Prochaska JJ (2020). Occupying multiple stigmatized identities: smoking and unemployment stigmas among the unemployed. SSM Popul Health.

[20] Prochaska J, Shi Y, Rogers A (2013). Tobacco use among the job-seeking unemployed in California. Prev Med.

[21] (2013). Results from the 2012 National Survey on Drug Use and Health: Summary of National Findings, NSDUH Series H-46, HHS Publication No. (SMA) 13-4795. Substance Abuse and Mental Health Services Administration.

[22] Study Randomizer.

[23] Schuck K, Otten R, Kleinjan M, Bricker JB, Engels RC (2014). Predictors of cessation treatment outcome and treatment moderators among smoking parents receiving quitline counselling or self-help material. Prev Med.

[24] Kendzor DE, Reitzel LR, Mazas CA, Cofta-Woerpel LM, Cao Y, Ji L, Costello TJ, Vidrine JI, Businelle MS, Li Y, Castro Y, Ahluwalia JS, Cinciripini PM, Wetter DW (2012). Individual- and area-level unemployment influence smoking cessation among African Americans participating in a randomized clinical trial. Soc Sci Med.

[25] Falba T, Teng HM, Sindelar JL, Gallo WT (2005). The effect of involuntary job loss on smoking intensity and relapse. Addiction.

[26] Weden MM, Astone NM, Bishai D (2006). Racial, ethnic, and gender differences in smoking cessation associated with employment and joblessness through young adulthood in the US. Soc Sci Med.

[27] Arcaya M, Glymour MM, Christakis NA, Kawachi I, Subramanian SV (2014). Individual and spousal unemployment as predictors of smoking and drinking behavior. Soc Sci Med.

[28] Kotz D, West R (2009). Explaining the social gradient in smoking cessation: it's not in the trying, but in the succeeding. Tob Control.

[29] Patnode CD, Henderson JT, Melnikow J, Coppola EL, Durbin S, Thomas R (2021). Interventions for Tobacco Cessation in Adults, Including Pregnant Women: An Evidence Update for the U.S. Preventive Services Task Force.

[30] Hartmann-Boyce J, Chepkin SC, Ye W, Bullen C, Lancaster T (2018). Nicotine replacement therapy versus control for smoking cessation. Cochrane Database Syst Rev.

[31] Krist AH, Davidson KW, Mangione CM, Barry MJ, Cabana M, Caughey AB, Donahue K, Doubeni CA, Epling JW, Kubik M, Ogedegbe G, Pbert L, Silverstein M, Simon MA, Tseng C, Wong JB, US Preventive Services Task Force (2021). Interventions for tobacco smoking cessation in adults, including pregnant persons: US preventive services task force recommendation statement. JAMA.

[32] Vinci C (2020). Cognitive behavioral and mindfulness-based interventions for smoking cessation: a review of the recent literature. Curr Oncol Rep.

[33] (2020). Smoking Cessation: A Report of the Surgeon General. Centers for Disease Control and Prevention.

[34] Williams GC, Niemiec CP, Patrick H, Ryan RM, Deci EL (2016). Outcomes of the Smoker's Health Project: a pragmatic comparative effectiveness trial of tobacco-dependence interventions based on self-determination theory. Health Educ Res.

[35] Hartmann-Boyce J, Livingstone-Banks J, Ordóñez-Mena JM, Fanshawe TR, Lindson N, Freeman SC, Sutton AJ, Theodoulou A, Aveyard P (2021). Behavioural interventions for smoking cessation: an overview and network meta-analysis. Cochrane Database Syst Rev.

[36] QuitGuide app. Smokefree Apps.

[37] Shahab L (2014). Why use CO-verified 4-week quit rates as the primary measure of stop smoking service success?. NHS Centre for Smoking Cessation and Training Briefings.

[38] Webb J, Peerbux S, Smittenaar P, Siddiqui S, Sherwani Y, Ahmed M, MacRae H, Puri H, Bhalla S, Majeed A (2020). Preliminary outcomes of a digital therapeutic intervention for smoking cessation in adult smokers: randomized controlled trial. JMIR Ment Health.

[39] Borland R, Yong HH, O'Connor RJ, Hyland A, Thompson ME (2010). The reliability and predictive validity of the Heaviness of Smoking Index and its two components: findings from the International Tobacco Control Four Country study. Nicotine Tob Res.

[40] Hall SM, Havassy BE, Wasserman DA (1990). Commitment to abstinence and acute stress in relapse to alcohol, opiates, and nicotine. J Consult Clin Psychol.

[41] Hall SM, Havassy BE, Wasserman DA (1991). Effects of commitment to abstinence, positive moods, stress, and coping on relapse to cocaine use. J Consult Clin Psychol.

[42] Spek V, Lemmens F, Chatrou M, van Kempen S, Pouwer F, Pop V (2013). Development of a smoking abstinence self-efficacy questionnaire. Int J Behav Med.

[43] Schneider D, Berent M, Thomas R, Krosnick J (2008). Measuring customer satisfaction and loyalty: improving the 'Net-Promoter' score. Van-haaften.

[44] Marler JD, Fujii CA, Utley DS, Tesfamariam LJ, Galanko JA, Patrick H (2019). Initial assessment of a comprehensive digital smoking cessation program that incorporates a mobile app, breath sensor, and coaching: cohort study. JMIR Mhealth Uhealth.

[45] Spiegelman D, Hertzmark E (2005). Easy SAS calculations for risk or prevalence ratios and differences. Am J Epidemiol.

[46] Berglund PA Using SAS® for Multiple Imputation and Analysis of Longitudinal Data. Paper 1738-2018. Institute for Social Research-University of Michigan.

[47] Iacoviello BM, Steinerman JR, Klein DB, Silver TL, Berger AG, Luo SX, Schork NJ (2017). Clickotine, a personalized smartphone app for smoking cessation: initial evaluation. JMIR Mhealth Uhealth.

[48] Garrison KA, Pal P, O'Malley SS, Pittman BP, Gueorguieva R, Rojiani R, Scheinost D, Dallery J, Brewer JA (2020). Craving to quit: a randomized controlled trial of smartphone app-based mindfulness training for smoking cessation. Nicotine Tob Res.

[49] Alessi SM, Rash CJ, Petry NM (2017). A randomized trial of adjunct mHealth abstinence reinforcement with transdermal nicotine and counseling for smoking cessation. Nicotine Tob Res.

[50] Baskerville NB, Struik LL, Guindon GE, Norman CD, Whittaker R, Burns C, Hammond D, Dash D, Brown KS (2018). Effect of a mobile phone intervention on quitting smoking in a young adult population of smokers: randomized controlled trial. JMIR Mhealth Uhealth.

[51] Danaher BG, Tyler MS, Crowley RC, Brendryen H, Seeley JR (2019). Outcomes and device usage for fully automated internet interventions designed for a smartphone or personal computer: the MobileQuit smoking cessation randomized controlled trial. J Med Internet Res.

[52] Piper ME, Cook JW, Schlam TR, Jorenby DE, Smith SS, Collins LM, Mermelstein R, Fraser D, Fiore MC, Baker TB (2018). A randomized controlled trial of an optimized smoking treatment delivered in primary care. Ann Behav Med.

[53] Herd N, Borland R, Hyland A (2009). Predictors of smoking relapse by duration of abstinence: findings from the International Tobacco Control (ITC) Four Country Survey. Addiction.

[54] Smit ES, Hoving C, Schelleman-Offermans K, West R, de Vries H (2014). Predictors of successful and unsuccessful quit attempts among smokers motivated to quit. Addict Behav.

[55] Prochaska JO, Norcross JC, Diclemente CC, Koocher GP, Norcross JC, Greene BA (2013). Applying the stages of change. Psychologists' Desk Reference. 3rd edition.

[56] Hughes JR (2000). Reduced smoking: an introduction and review of the evidence. Addiction.

[57] Begh R, Lindson-Hawley N, Aveyard P (2015). Does reduced smoking if you can't stop make any difference?. BMC Med.

[58] Bricker JB, Levin M, Lappalainen R, Mull K, Sullivan B, Santiago-Torres M (2021). Mechanisms of smartphone apps for cigarette smoking cessation: results of a serial mediation model from the iCanQuit randomized trial. JMIR Mhealth Uhealth.

[59] Benowitz NL, Bernert JT, Foulds J, Hecht SS, Jacob P, Jarvis MJ, Joseph A, Oncken C, Piper ME (2020). Biochemical verification of tobacco use and abstinence: 2019 update. Nicotine Tob Res.

[60] Eysenbach G, CONSORT-EHEALTH Group (2011). CONSORT-EHEALTH: improving and standardizing evaluation reports of Web-based and mobile health interventions. J Med Internet Res.

