# Supplementary material for: Outcomes of a Comprehensive Mobile Smoking Cessation Program With Nicotine Replacement Therapy in Adult Smokers: Pilot Randomized Controlled Trial
Source: JMIR Mhealth Uhealth. 2022 Nov 24;10(11):e41658. doi: 10.2196/41658 (PMC9732762; doi:10.2196/41658)
Supplement: Multimedia Appendix 1 [file mhealth_v10i11e41658_app1.docx]

Multimedia Appendix 1. Participant Feedback

| Question | All  n (%) | Pivot  n (%) | QuitGuide  n (%) | *P-*value |
| --- | --- | --- | --- | --- |
| How has using your assigned program affected your motivation to quit smoking? (week 2) | n=184 | n=92 | n=92 | .005 |
| increased | 126 (68.5%) | 72 (78.3%) | 54 (58.7%) |  |
| not affected / decreased^a^ | 58 (31.5%) | 20 (21.8%) | 38 (41.3%) |  |
| How has using your assigned program affected the number of cigarettes you smoke per day?  (week 2) | n=184 | n=92 | n=92 | .37 |
| increased | 4 (2.2%) | 2 (2.2%) | 2 (2.2%) |  |
| not affected | 59 (32.1%) | 25 (27.2%) | 34 (37.0%) |  |
| decreased | 121 (65.8%) | 65 (70.7%) | 56 (60.9%) |  |
| Which of the following best describes your need for your assigned program? (week 3) | n=179 | n=89 | n=90 | .04 |
| I really need this program | 139 (77.7%) | 75 (84.3%) | 64 (71.1%) |  |
| I somewhat need this program /  I don't need this program /  I am not at all interested in this program^b^ | 40 (22.3%) | 14 (15.7%) | 26 (28.9%) |  |
| Which of the following best describes your thoughts on your program? (week 3) | n=179 | n=89 | n=90 | .045 |
| Nothing else can help me with smoking | 7 (3.9%) | 3 (3.4%) | 4 (4.4%) |  |
| Among the tools that can help me with smoking, this one can help me the most | 76 (42.5%) | 49 (55.1%) | 27 (30.0%) |  |
| Among the tools that can help me with smoking, this one can help me some | 52 (29.1%) | 22 (24.7%) | 30 (33.3%) |  |
| I think it might help me with smoking | 35 (19.6%) | 14 (15.7%) | 21 (23.2%) |  |
| I don’t think it will help me with smoking | 6 (3.4%) | 1 (1.1%) | 5 (5.6%) |  |
| It will not help me with smoking | 3 (1.7%) | 0 (0.0%) | 3 (3.3%) |  |
| Which of the following best describes your assigned program’s ability to help someone quit smoking? (week 3) | n=179 | n=89 | n=90 | .003 |
| extremely helpful to quitting smoking | 56 (31.3%) | 35 (39.3%) | 21 (23.3%) |  |
| helpful to quitting smoking | 109 (60.9%) | 53 (59.6%) | 56 (62.2%) |  |
| does not affect being able to quit smoking | 13 (7.3%) | 1 (1.1%) | 12 (13.3%) |  |
| makes quitting smoking harder | 1 (0.6%) | 0 (0.0%) | 1 (1.1%) |  |
| Which of the following best describes what you have learned from using your program? (week 3) | n=179 | n=89 | n=90 | .001 |
| I’ve learned some really unique/key insights into my smoking behavior | 123 (68.7%) | 72 (80.0%) | 51 (56.7%) |  |
| I’ve learned some things but nothing that impactful | 42 (23.5%) | 16 (18.0%) | 26 (28.9%) |  |
| I have not learned anything from the program /  I am more confused after using the program^c^ | 14 (7.8%) | 1 (1.1%) | 13 (14.4%) |  |

^a^  not affected: Pivot 19 (20.7%), QuitGuide 35 (38.0%) 
decreased: Pivot 1 (1.1%), QuitGuide 3 (3.3%)

^b^  I somewhat need this program: Pivot: 14 (15.7%), QuitGuide: 21 (23.3%);  
I don't need this program: Pivot: 0 (0%), QuitGuide: 2 (2.2%);  
I am not at all interested in this program: Pivot: 0 (0%), QuitGuide: 3 (3.3%);

^c^ I have not learned anything from the program: Pivot 1 (1.1%), QuitGuide 12 (13.3%) 
I am more confused after using the program: Pivot 0 (0.0%), QuitGuide 1 (1.1%) 
