# Supplementary material for: Outcomes of a Comprehensive Mobile Smoking Cessation Program With Nicotine Replacement Therapy in Adult Smokers: Pilot Randomized Controlled Trial
Source: JMIR Mhealth Uhealth. 2022 Nov 24;10(11):e41658. doi: 10.2196/41658 (PMC9732762; doi:10.2196/41658)
Supplement: Multimedia Appendix 2 [file mhealth_v10i11e41658_app2.docx]

Multimedia Appendix 2. NPS at 4, 12, and 26 weeks

| Outcome | Cohort | n | NPS^a^ | Promoter  (%) | Neutral (%) | Detractor (%) | *P*-value ^b^ |
| --- | --- | --- | --- | --- | --- | --- | --- |
| 4 weeks | Pivot | 91 | 50.6 | 63.7 | 23.1 | 13.2 | <0.001 |
|  | QuitGuide | 88 | 1.1 | 39.8 | 21.6 | 38.6 |  |
| 12 weeks | Pivot | 92 | 44.6 | 59.8 | 25.0 | 15.2 | 0.02 |
|  | QuitGuide | 91 | 11.0 | 44.0 | 23.1 | 33.0 |  |
| 26 weeks | Pivot | 90 | 57.8 | 71.1 | 15.6 | 13.3 | 0.02 |
|  | QuitGuide | 89 | 23.6 | 52.8 | 18.0 | 29.2 |  |

^a^ NPS formula: % Promoter - % Detractor

^b^ Multinomial regression on classification (Promoter, Neutral, Detractor) adjusted for randomization covariates
