# Supplementary material for: Outcomes of a Comprehensive Mobile Smoking Cessation Program With Nicotine Replacement Therapy in Adult Smokers: Pilot Randomized Controlled Trial
Source: JMIR Mhealth Uhealth. 2022 Nov 24;10(11):e41658. doi: 10.2196/41658 (PMC9732762; doi:10.2196/41658)
Supplement: Multimedia Appendix 3 [file mhealth_v10i11e41658_app3.pdf]

# CONSORT-EHEALTH (V 1.6.1) - Submission/Publication Form

The CONSORT-EHEALTH checklist is intended for authors of randomized trials evaluating web-based and Internet-based applications/interventions, including mobile interventions, electronic games (incl multiplayer games), social media, certain telehealth applications, and other interactive and/or networked electronic applications. Some of the items (e.g. all subitems under item 5 - description of the intervention) may also be applicable for other study designs.

The goal of the CONSORT EHEALTH checklist and guideline is to be
a) a guide for reporting for authors of RCTs,
b) to form a basis for appraisal of an ehealth trial (in terms of validity)

CONSORT-EHEALTH items/subitems are MANDATORY reporting items for studies published in the Journal of Medical Internet Research and other journals / scientific societies endorsing the checklist.

Items numbered 1., 2., 3., 4a., 4b etc are original CONSORT or CONSORT-NPT (non-pharmacologic treatment) items.
Items with Roman numerals (i., ii, iii, iv etc.) are CONSORT-EHEALTH extensions/clarifications.

As the CONSORT-EHEALTH checklist is still considered in a formative stage, we would ask that you also RATE ON A SCALE OF 1-5 how important/useful you feel each item is FOR THE PURPOSE OF THE CHECKLIST and reporting guideline (optional).

Mandatory reporting items are marked with a red *.
In the textboxes, either copy & paste the relevant sections from your manuscript into this form - please include any quotes from your manuscript in QUOTATION MARKS,
or answer directly by providing additional information not in the manuscript, or elaborating on why the item was not relevant for this study.

YOUR ANSWERS WILL BE PUBLISHED AS  A SUPPLEMENTARY FILE TO YOUR PUBLICATION IN JMIR AND ARE CONSIDERED PART OF YOUR PUBLICATION (IF ACCEPTED).
Please fill in these questions diligently. Information will not be copyedited, so please use proper spelling and grammar, use correct capitalization, and avoid abbreviations.

DO NOT FORGET TO SAVE AS PDF _AND_ CLICK THE SUBMIT BUTTON SO YOUR ANSWERS ARE IN OUR DATABASE !!!

Citation Suggestion (if you append the pdf as Appendix we suggest to cite this paper in the caption):
Eysenbach G, CONSORT-EHEALTH Group

CONSORT-EHEALTH: Improving and Standardizing Evaluation Reports of Web-based and Mobile Health Interventions
J Med Internet Res 2011;13(4):e126
URL: http://www.jmir.org/2011/4/e126/
doi: 10.2196/jmir.1923
PMID: 22209829

**marler@carrot.co** (not shared) Switch account

Draft not saved

* Required

Your name *
First Last

Jennifer Marler

Primary Affiliation (short), City, Country *
University of Toronto, Toronto, Canada

Pivot Health Technologies Inc.

Your e-mail address *
abc@gmail.com

marler@pivot.co

Title of your manuscript *

Provide the (draft) title of your manuscript.

Outcomes of a Comprehensive Mobile Smoking Cessation Program with Nicotine Replacement Therapy in Adult Smokers: Pilot Randomized Controlled Trial

Name of your App/Software/Intervention *

If there is a short and a long/alternate name, write the short name first and add the long name in brackets.

Pivot [Pivot Journey - Quit Smoking]

Evaluated Version (if any)

e.g. "V1", "Release 2017-03-01", "Version 2.0.27913"

Your answer

Language(s) *

What language is the intervention/app in? If multiple languages are available, separate by comma (e.g. "English, French")

English

URL of your Intervention Website or App

e.g. a direct link to the mobile app on app in appstore (itunes, Google Play), or URL of the website. If the intervention is a DVD or hardware, you can also link to an Amazon page.

https://apple.com/us/app/pivot-journey-quit-smoking/id1236893700 https://pivot.co/ http[illegible]

URL of an image/screenshot (optional)

Your answer

Accessibility *

Can an enduser access the intervention presently?

- ○ access is free and open
- ○ access only for special usergroups, not open
- ◉ access is open to everyone, but requires payment/subscription/in-app purchases
- ○ app/intervention no longer accessible
- ○ Other:

Primary Medical Indication/Disease/Condition *

e.g. "Stress", "Diabetes", or define the target group in brackets after the condition, e.g. "Autism (Parents of children with)", "Alzheimers (Informal Caregivers of)"

Smoking cessation

Primary Outcomes measured in trial *

comma-separated list of primary outcomes reported in the trial

Engagement via total self-reported app openin

Secondary/other outcomes

Are there any other outcomes the intervention is expected to affect?

"Outcomes included engagement and retention, attitudes towards quitting smoking, smoking behavior, and participant feedback...Cessation outcomes included self-reported 7- and 30-day point prevalence abstinence (PPA), abstinence from all tobacco products and continuous abstinence at 12 and 26 weeks. PPA and continuous abstinence were biovalidated via breath CO samples."

Recommended "Dose" *

What do the instructions for users say on how often the app should be used?

- ( ) Approximately Daily
- ( ) Approximately Weekly
- ( ) Approximately Monthly
- ( ) Approximately Yearly
- (x) "as needed"
- ( ) Other:

Approx. Percentage of Users (starters) still using the app as recommended after 3 months *

- (x) unknown / not evaluated
- ( ) 0-10%
- ( ) 11-20%
- ( ) 21-30%
- ( ) 31-40%
- ( ) 41-50%
- ( ) 51-60%
- ( ) 61-70%
- ( ) 71%-80%
- ( ) 81-90%
- ( ) 91-100%
- ( ) Other:

Overall, was the app/intervention effective? *

- (x) yes: all primary outcomes were significantly better in intervention group vs control
- ( ) partly: SOME primary outcomes were significantly better in intervention group vs control
- ( ) no statistically significant difference between control and intervention
- ( ) potentially harmful: control was significantly better than intervention in one or more outcomes
- ( ) inconclusive: more research is needed
- ( ) Other:

Article Preparation Status/Stage *

At which stage in your article preparation are you currently (at the time you fill in this form)

- ○ not submitted yet - in early draft status
- ○ not submitted yet - in late draft status, just before submission
- ○ submitted to a journal but not reviewed yet
- ◉ submitted to a journal and after receiving initial reviewer comments
- ○ submitted to a journal and accepted, but not published yet
- ○ published
- ○ Other:

Journal *

If you already know where you will submit this paper (or if it is already submitted), please provide the journal name (if it is not JMIR, provide the journal name under "other")

- ○ not submitted yet / unclear where I will submit this
- ○ Journal of Medical Internet Research (JMIR)
- ◉ JMIR mHealth and UHealth
- ○ JMIR Serious Games
- ○ JMIR Mental Health
- ○ JMIR Public Health
- ○ JMIR Formative Research
- ○ Other JMIR sister journal
- ○ Other:

Is this a full powered effectiveness trial or a pilot/feasibility trial? *

- (selected) Pilot/feasibility
- Fully powered

Manuscript tracking number *

If this is a JMIR submission, please provide the manuscript tracking number under "other" (The ms tracking number can be found in the submission acknowledgement email, or when you login as author in JMIR. If the paper is already published in JMIR, then the ms tracking number is the four-digit number at the end of the DOI, to be found at the bottom of each published article in JMIR)

- no ms number (yet) / not (yet) submitted to / published in JMIR
- (selected) Other: ms #41658

TITLE AND ABSTRACT

1a) TITLE: Identification as a randomized trial in the title

1a) Does your paper address CONSORT item 1a? *

I.e does the title contain the phrase "Randomized Controlled Trial"? (if not, explain the reason under "other")

- (selected) yes
- Other:

### 1a-i) Identify the mode of delivery in the title

Identify the mode of delivery. Preferably use “web-based” and/or “mobile” and/or “electronic game” in the title. Avoid ambiguous terms like “online”, “virtual”, “interactive”. Use “Internet-based” only if Intervention includes non-web-based Internet components (e.g. email), use “computer-based” or “electronic” only if offline products are used. Use “virtual” only in the context of “virtual reality” (3-D worlds). Use “online” only in the context of “online support groups”. Complement or substitute product names with broader terms for the class of products (such as “mobile” or “smart phone” instead of “iphone”), especially if the application runs on different platforms.

| | 1 | 2 | 3 | 4 | 5 | |
|---|---|---|---|---|---|---|
| subitem not at all important | ○ | ○ | ○ | ◉ | ○ | essential |

Clear selection

### Does your paper address subitem 1a-i? *

Copy and paste relevant sections from manuscript title (include quotes in quotation marks "like this" to indicate direct quotes from your manuscript), or elaborate on this item by providing additional information not in the ms, or briefly explain why the item is not applicable/relevant for your study

"Outcomes of a Comprehensive Mobile Smoking Cessation Program with Nicotine Replacement Therapy in Adult Smokers: Pilot Randomized Controlled Trial"

### 1a-ii) Non-web-based components or important co-interventions in title

Mention non-web-based components or important co-interventions in title, if any (e.g., “with telephone support”).

| | 1 | 2 | 3 | 4 | 5 | |
|---|---|---|---|---|---|---|
| subitem not at all important | ○ | ○ | ◉ | ○ | ○ | essential |

Clear selection

Does your paper address subitem 1a-ii?

Copy and paste relevant sections from manuscript title (include quotes in quotation marks "like this" to indicate direct quotes from your manuscript), or elaborate on this item by providing additional information not in the ms, or briefly explain why the item is not applicable/relevant for your study

"Outcomes of a Comprehensive Mobile Smoking Cessation Program with Nicotine Replacement Therapy in Adult Smokers: Pilot Randomized Controlled Trial"

1a-iii) Primary condition or target group in the title

Mention primary condition or target group in the title, if any (e.g., “for children with Type I Diabetes”) Example: A Web-based and Mobile Intervention with Telephone Support for Children with Type I Diabetes: Randomized Controlled Trial

| | 1 | 2 | 3 | 4 | 5 | |
|---|---|---|---|---|---|---|
| subitem not at all important | ○ | ○ | ○ | ◉ | ○ | essential |

Clear selection

Does your paper address subitem 1a-iii? *

Copy and paste relevant sections from manuscript title (include quotes in quotation marks "like this" to indicate direct quotes from your manuscript), or elaborate on this item by providing additional information not in the ms, or briefly explain why the item is not applicable/relevant for your study

"Outcomes of a Comprehensive Mobile Smoking Cessation Program with Nicotine Replacement Therapy in Adult Smokers..."

1b) ABSTRACT: Structured summary of trial design, methods, results, and conclusions

NPT extension: Description of experimental treatment, comparator, care providers, centers, and blinding status.

1b-i) Key features/functionalities/components of the intervention and comparator in the METHODS section of the ABSTRACT

Mention key features/functionalities/components of the intervention and comparator in the abstract. If possible, also mention theories and principles used for designing the site. Keep in mind the needs of systematic reviewers and indexers by including important synonyms. (Note: Only report in the abstract what the main paper is reporting. If this information is missing from the main body of text, consider adding it)

| | 1 | 2 | 3 | 4 | 5 | |
|---|---|---|---|---|---|---|
| subitem not at all important | ○ | ○ | ◉ | ○ | ○ | essential |

Clear selection

Does your paper address subitem 1b-i? *

Copy and paste relevant sections from the manuscript abstract (include quotes in quotation marks "like this" to indicate direct quotes from your manuscript), or elaborate on this item by providing additional information not in the ms, or briefly explain why the item is not applicable/relevant for your study

"Pivot, a US Clinical Practice Guideline (USCPG)-based mobile smoking cessation program, comprises a personal carbon monoxide (CO) breath sensor, smartphone app, in-app, text-based human-provided coaching, nicotine replacement therapy (NRT), and moderated online community."..."QuitGuide, a USCPG-based smoking cessation smartphone app from the National Cancer Institute (NCI)."

### 1b-ii) Level of human involvement in the METHODS section of the ABSTRACT

Clarify the level of human involvement in the abstract, e.g., use phrases like “fully automated” vs. “therapist/nurse/care provider/physician-assisted” (mention number and expertise of providers involved, if any). (Note: Only report in the abstract what the main paper is reporting. If this information is missing from the main body of text, consider adding it)

| | 1 | 2 | 3 | 4 | 5 | |
|---|---|---|---|---|---|---|
| subitem not at all important | ○ | ○ | ◉ | ○ | ○ | essential |

Clear selection

### Does your paper address subitem 1b-ii?

Copy and paste relevant sections from the manuscript abstract (include quotes in quotation marks "like this" to indicate direct quotes from your manuscript), or elaborate on this item by providing additional information not in the ms, or briefly explain why the item is not applicable/relevant for your study

"Pivot, a US Clinical Practice Guideline (USCPG)-based mobile smoking cessation program, comprises a personal carbon monoxide (CO) breath sensor, smartphone app, in-app, text-based human-provided coaching..."

1b-iii) Open vs. closed, web-based (self-assessment) vs. face-to-face assessments in the METHODS section of the ABSTRACT

Mention how participants were recruited (online vs. offline), e.g., from an open access website or from a clinic or a closed online user group (closed usergroup trial), and clarify if this was a purely web-based trial, or there were face-to-face components (as part of the intervention or for assessment). Clearly say if outcomes were self-assessed through questionnaires (as common in web-based trials). Note: In traditional offline trials, an open trial (open-label trial) is a type of clinical trial in which both the researchers and participants know which treatment is being administered. To avoid confusion, use “blinded” or “unblinded” to indicated the level of blinding instead of “open”, as “open” in web-based trials usually refers to “open access” (i.e. participants can self-enrol). (Note: Only report in the abstract what the main paper is reporting. If this information is missing from the main body of text, consider adding it)

| | 1 | 2 | 3 | 4 | 5 | |
|---|---|---|---|---|---|---|
| subitem not at all important | ○ | ○ | ◉ | ○ | ○ | essential |

Clear selection

Does your paper address subitem 1b-iii?

Copy and paste relevant sections from the manuscript abstract (include quotes in quotation marks "like this" to indicate direct quotes from your manuscript), or elaborate on this item by providing additional information not in the ms, or briefly explain why the item is not applicable/relevant for your study

"In this remote pilot randomized controlled trial (RCT), cigarette smokers in the US were recruited online and randomized to Pivot or QuitGuide...Data were self-reported via weekly online questionnaires for 12 weeks and at 26 weeks..."

### 1b-iv) RESULTS section in abstract must contain use data

Report number of participants enrolled/assessed in each group, the use/uptake of the intervention (e.g., attrition/adherence metrics, use over time, number of logins etc.), in addition to primary/secondary outcomes. (Note: Only report in the abstract what the main paper is reporting. If this information is missing from the main body of text, consider adding it)

| | 1 | 2 | 3 | 4 | 5 | |
|---|---|---|---|---|---|---|
| subitem not at all important | ○ | ○ | ◉ | ○ | ○ | essential |

Clear selection

### Does your paper address subitem 1b-iv?

Copy and paste relevant sections from the manuscript abstract (include quotes in quotation marks "like this" to indicate direct quotes from your manuscript), or elaborate on this item by providing additional information not in the ms, or briefly explain why the item is not applicable/relevant for your study

Participants comprised 188 smokers (94 Pivot, 94 QuitGuide): mean (SD) age 46.4 (9.2) years, 104 women (55.3%), 128 White individuals (68.1%), mean (SD) cigarettes per day (CPD) 17.6 (9.0). Engagement via mean (SD) total app openings through 12 weeks (primary outcome) was Pivot 157.9 (SD 210.6) vs. QuitGuide 86.5 (SD 66.3) (incidence rate ratio [IRR],1.8; 95% CI, 1.4, 2.3; P<.001)."

### 1b-v) CONCLUSIONS/DISCUSSION in abstract for negative trials

Conclusions/Discussions in abstract for negative trials: Discuss the primary outcome - if the trial is negative (primary outcome not changed), and the intervention was not used, discuss whether negative results are attributable to lack of uptake and discuss reasons. (Note: Only report in the abstract what the main paper is reporting. If this information is missing from the main body of text, consider adding it)

| | 1 | 2 | 3 | 4 | 5 | |
|---|---|---|---|---|---|---|
| subitem not at all important | ○ | ○ | ○ | ○ | ○ | essential |

Does your paper address subitem 1b-v?

Copy and paste relevant sections from the manuscript abstract (include quotes in quotation marks "like this" to indicate direct quotes from your manuscript), or elaborate on this item by providing additional information not in the ms, or briefly explain why the item is not applicable/relevant for your study

"In this RCT comparing the app-based smoking cessation programs Pivot and QuitGuide, Pivot participants had higher engagement and biovalidated cessation rates, and more favorable user feedback at 12 and 26 weeks."

INTRODUCTION

2a) In INTRODUCTION: Scientific background and explanation of rationale

2a-i) Problem and the type of system/solution

Describe the problem and the type of system/solution that is object of the study: intended as stand-alone intervention vs. incorporated in broader health care program? Intended for a particular patient population? Goals of the intervention, e.g., being more cost-effective to other interventions, replace or complement other solutions? (Note: Details about the intervention are provided in “Methods” under 5)

| | 1 | 2 | 3 | 4 | 5 | |
|---|---|---|---|---|---|---|
| subitem not at all important | ○ | ○ | ◉ | ○ | ○ | essential |

Clear selection

Does your paper address subitem 2a-i? *

Copy and paste relevant sections from the manuscript (include quotes in quotation marks "like this" to indicate direct quotes from your manuscript), or elaborate on this item by providing additional information not in the ms, or briefly explain why the item is not applicable/relevant for your study

"Tobacco is responsible for over 8 million deaths around the world per year. On its own, smoking is a leading cause of preventable illness and death globally [1]. Despite this, most quit attempts are undertaken without assistance and are unsuccessful [2].

In recent years, mobile app-based programs for smoking cessation have become prevalent and show promise with greater accessibility than traditional face-to-face programs. A variety of these programs currently exist, but many lack evidence of their efficacy...

A cohort study of the Pivot program was published in 2021. During the study, Pivot included a mobile app, personal CO breath sensor, and text-based human coaching. At 3 months post-program completion (mean 7.2 months after enrollment), 32.0% (ITT) and 37.5% (Completer) of participants achieved 7-day PPA; 27.6% (ITT) and 32.4% (Completer) reported 30-day PPA [15]. The Pivot program has since undergone updates, and now includes access to NRT and a moderated online community. These changes, the need for long-term results for app-based cessation programs, and the ongoing need to assess the performance of Pivot within the context of current smoking cessation programs, warrant new investigation of the Pivot program. The primary aim of the study is to compare user engagement and retention in the Pivot smoking cessation program to the current mobile standard of care. The secondary aims are to compare changes in attitudes towards quitting smoking, changes in smoking behavior and feedback on the user experience."

### 2a-ii) Scientific background, rationale: What is known about the (type of) system

Scientific background, rationale: What is known about the (type of) system that is the object of the study (be sure to discuss the use of similar systems for other conditions/diagnoses, if appropiate), motivation for the study, i.e. what are the reasons for and what is the context for this specific study, from which stakeholder viewpoint is the study performed, potential impact of findings [2]. Briefly justify the choice of the comparator.

| | 1 | 2 | 3 | 4 | 5 | |
|---|---|---|---|---|---|---|
| subitem not at all important | ○ | ○ | ◉ | ○ | ○ | essential |

Clear selection

Does your paper address subitem 2a-ii? *

Copy and paste relevant sections from the manuscript (include quotes in quotation marks "like this" to indicate direct quotes from your manuscript), or elaborate on this item by providing additional information not in the ms, or briefly explain why the item is not applicable/relevant for your study

"In recent years, mobile app-based programs for smoking cessation have become prevalent and show promise with greater accessibility than traditional face-to-face programs. A variety of these programs currently exist, but many lack evidence of their efficacy. A 2019 meta-analysis by Whittaker et al. analyzed 5 studies and found no evidence that smartphone app cessation programs improved smoking cessation outcomes when compared to lower-intensity cessation apps or minimal non-app support (relative risk ratio [RR] 1.00; 95% CI, 0.66, 1.52; I2 = 59%) [3]. This finding was of low certainty, however, due to inconsistencies and imprecision, highlighting the need for more RCTs of app-based cessation programs.

Bricker et al. compared two app-based cessation programs in a 2020 RCT. At 12 months, participants randomized to iCanQuit, an acceptance and commitment therapy (ACT)-based smoking cessation app, had 1.49 times higher odds of quitting smoking than participants randomized to QuitGuide, a USCPG-based smoking cessation app [4]. Previously, in 2014, Bricker et al. ran a similar RCT comparing SmartQuit, another ACT-based smoking cessation app, with QuitGuide. At 2 months, 13% of SmartQuit and 8% of QuitGuide participants quit smoking (OR, =2.7; 95% CI,=0.8,-10.3) [5]. Another RCT, by BinDhim et al. in 2018, compared a smoking cessation decision-aid app with an information-only control app. At 6 months, 10.2% using the decision-aid app and 4.8% using the control self-reported continuous abstinence from smoking (RR 2.02; 95% CI, 1.08, 3.81) [6].

More comprehensive programs with nicotine replacement therapy (NRT) and additional support have also been studied. In a 2020 RCT, Webb et al. compared a cognitive behavioral therapy (CBT)-based smoking cessation app with one-on-one coaching (Quit Genius), to very brief advice (VBA). All participants had access to 3 months of NRT and a random half of each arm received a carbon monoxide (CO) breath sensor device. At 52 weeks, 34.7% (92/265) of participants in the treatment arm achieved 7-day PPA vs. 29.4% (78/265) in the control (RR, 1.20;, 95% CI, 0.94, 1.54). The assignment of the CO breath sensor device, or lack thereof, did not significantly predict whether a participant achieved 7-day PPA [7]. Tweet2Quit, a program including an app, text messages, and a Twitter group, was compared to a non-app control in a 2016 RCT by Pechmann et al. Both groups received 56 days of NRT patches, instruction to set a quit date, and referral to smokefree.gov. At 60 days, the Tweet2Quit arm had 40% smoking abstinence compared to 20% among controls [8].

Technology-enabled features of smoking cessation programs, including CO breath sensors, online communities and SMS-based coaching have been explored previously. In The Tobacco Dependence Treatment Handbook: A Guide to Best Practices [9] the authors reported that, “providing individualized feedback about changes in personal levels of carbon monoxide before and after smoking is a powerful message that encourages individuals to make a quit attempt”, demonstrating the utility of CO monitors for smoking cessation. Beard et al. [10] provided smokers not seeking out a quit smoking program with personal CO breath sensors for 6 weeks, with a goal to maintain their CO level below 10 parts per million

(ppm). Participants were not instructed to quit. The 10 participants used the CO monitors an average of 3 times a day, decreased their average daily cigarette consumption from 14.1 (SD 6.03) at baseline to 9.8 (SD 4.95; P=.036) during the 2 weeks of daily CO monitoring and to 9.5 (SD 5.50; P=.127) at 6-week follow-up. At follow-up, 50% (5/10) of participants had attempted to quit smoking and one successfully quit. The majority of the participants reported the CO monitor was helpful (79.3%, n=111/140 responses) and that they felt as though the monitor had reduced their cigarette consumption (70%, 7/10 participants). Beard et al. concluded that the use of the CO monitors increased motivation to consider a quit attempt. A 2020 cohort study also assessed the use of a personal CO breath sensor, specifically the Pivot Breath Sensor, by 234 adult smokers. The sensor's impact on attitudes toward quitting smoking and smoking behavior was investigated over 12 weeks. Participants in this study had a significant (P<.001) increase in motivation to quit smoking, 28.2% (66/234) made at least one quit attempt, and 38.5% (90/234) reduced the number of cigarettes smoked per day at 12 weeks [11].

Smoking cessation programs with online communities have also been studied. Graham et al. [12] conducted a propensity score weighting of The iQUITT Study, an RCT of telephone and internet treatment for smoking cessation where the Internet arm of the study included a large and well-established online community. Of the 492 participants assigned to the iQUITT study's Internet arm, 40.2% (198/492) did not engage with the online community, 37.4% (184/492) engaged both actively and passively, and 22.4% (110/492) engaged only passively. At 3 months, Average Treatment Effects weighted abstinence rates were 4.2% for those that did not use the online community, 15.1% for those that used the online community passively, and 20.4% for those that used the online community both passively and actively. Users of the online community were also more likely to quit smoking than nonusers. Sadasivam et al. [13] conducted a study testing the functions of Decide2Quit.org, a web-based tobacco intervention that contains an online community, messaging with tobacco treatment specialists, and other major functions to support tobacco cessation. In bivariate comparison among 204 smokers, the online community had a positive association with quit outcomes at 6 months, and the highest differential in quit outcomes for those that used the function compared with other functions of the online quit program. Messaging with tobacco treatment specialists was negatively associated with quit outcomes at 6 months, however the authors suggest these results could be confounded by those utilizing the specialists as having the most difficulty quitting smoking.

Studies focused on the impact of one-on-one text coaching or messaging with tobacco treatment specialists are limited. Sadasivam et al. [14] conducted a secondary analysis of a web-based smoking cessation intervention that includes asynchronous messaging with trained tobacco treatment specialists. The goal of the study was to evaluate the association of this communication with smoking cessation during a period of 6 months. Of the 725 smokers in the study, 33.8% (245/725) messaged a tobacco treatment specialist at least once. The amount of messaging with a tobacco treatment specialist had no association with cessation outcomes at 6 months, although the authors suggest low engagement or lack of power to be explanations for the lack of association found.

A cohort study of the Pivot program was published in 2021. During the study, Pivot included a mobile app, personal CO breath sensor, and text-based human coaching. At 3 months post-program completion (mean 7.2 months after enrollment), 32.0% (ITT) and 37.5% (Completer) of participants achieved 7-day PPA; 27.6% (ITT) and 32.4% (Completer)

reported 30-day PPA [15]. The Pivot program has since undergone updates, and now includes access to NRT and a moderated online community. These changes, the need for long-term results for app-based cessation programs, and the ongoing need to assess the performance of Pivot within the context of current smoking cessation programs, warrant new investigation of the Pivot program. The primary aim of the study is to compare user engagement and retention in the Pivot smoking cessation program to the current mobile standard of care. The secondary aims are to compare changes in attitudes towards quitting smoking, changes in smoking behavior and feedback on the user experience...

...QuitGuide was used as the control for the following reasons: the content follows the USCPG for tobacco cessation, it is an app-based smoking cessation program thereby enabling intra-study comparison of same-modality interventions, the app is non-proprietary and is free to the public, and its use in previous well-designed RCTs [4,5], provides context and enables inter-study comparison to earlier data."

## 2b) In INTRODUCTION: Specific objectives or hypotheses

### Does your paper address CONSORT subitem 2b? *

Copy and paste relevant sections from the manuscript (include quotes in quotation marks "like this" to indicate direct quotes from your manuscript), or elaborate on this item by providing additional information not in the ms, or briefly explain why the item is not applicable/relevant for your study

"The primary aim of the study is to compare user engagement and retention in the Pivot smoking cessation program to the current mobile standard of care. The secondary aims are to compare changes in attitudes towards quitting smoking, changes in smoking behavior and feedback on the user experience."

## METHODS

3a) Description of trial design (such as parallel, factorial) including allocation ratio

Does your paper address CONSORT subitem 3a? *

Copy and paste relevant sections from the manuscript (include quotes in quotation marks "like this" to indicate direct quotes from your manuscript), or elaborate on this item by providing additional information not in the ms, or briefly explain why the item is not applicable/relevant for your study

"In this two-arm, parallel-group, non-crossover, single-center, individually-randomized controlled trial, participants were randomized to one of two app-based smoking cessation programs: QuitGuide (control) or Pivot (intervention)."

3b) Important changes to methods after trial commencement (such as eligibility criteria), with reasons

Does your paper address CONSORT subitem 3b? *

Copy and paste relevant sections from the manuscript (include quotes in quotation marks "like this" to indicate direct quotes from your manuscript), or elaborate on this item by providing additional information not in the ms, or briefly explain why the item is not applicable/relevant for your study

There were no important changes to methods after trial commencement.

3b-i) Bug fixes, Downtimes, Content Changes

Bug fixes, Downtimes, Content Changes: ehealth systems are often dynamic systems. A description of changes to methods therefore also includes important changes made on the intervention or comparator during the trial (e.g., major bug fixes or changes in the functionality or content) (5-iii) and other “unexpected events” that may have influenced study design such as staff changes, system failures/downtimes, etc. [2].

| | 1 | 2 | 3 | 4 | 5 | |
|---|---|---|---|---|---|---|
| subitem not at all important | ○ | ○ | ◉ | ○ | ○ | essential |

Clear selection

Does your paper address subitem 3b-i?

Copy and paste relevant sections from the manuscript (include quotes in quotation marks "like this" to indicate direct quotes from your manuscript), or elaborate on this item by providing additional information not in the ms, or briefly explain why the item is not applicable/relevant for your study

There were no major bug fixes, downtimes or changes in the functionality or content, or other 'unexpected events such as staff changes or system failures that may have influenced study design or conduct.

4a) Eligibility criteria for participants

Does your paper address CONSORT subitem 4a? *

Copy and paste relevant sections from the manuscript (include quotes in quotation marks "like this" to indicate direct quotes from your manuscript), or elaborate on this item by providing additional information not in the ms, or briefly explain why the item is not applicable/relevant for your study

"Eligibility criteria included the following: 21+ years of age, current daily cigarette smoker (≥ 5 CPD) for the past 12 months, plans to quit smoking in the next 30 days, resident of the United States, able to read and comprehend English, owns and uses a smartphone compatible with the study app (iPhone 5 and above with operating system iOS 12 and above, or, Android 7.0 and above with operating system Android 7.0 and above), has daily internet access on smartphone, and self-reported comfort with downloading and using smartphone apps."

4a-i) Computer / Internet literacy

Computer / Internet literacy is often an implicit "de facto" eligibility criterion - this should be explicitly clarified.

| | 1 | 2 | 3 | 4 | 5 | |
|---|---|---|---|---|---|---|
| subitem not at all important | ○ | ● | ○ | ○ | ○ | essential |

Clear selection

Does your paper address subitem 4a-i?

Copy and paste relevant sections from the manuscript (include quotes in quotation marks "like this" to indicate direct quotes from your manuscript), or elaborate on this item by providing additional information not in the ms, or briefly explain why the item is not applicable/relevant for your study

"...and self-reported comfort with downloading and using smartphone apps."

4a-ii) Open vs. closed, web-based vs. face-to-face assessments:

Open vs. closed, web-based vs. face-to-face assessments: Mention how participants were recruited (online vs. offline), e.g., from an open access website or from a clinic, and clarify if this was a purely web-based trial, or there were face-to-face components (as part of the intervention or for assessment), i.e., to what degree got the study team to know the participant. In online-only trials, clarify if participants were quasi-anonymous and whether having multiple identities was possible or whether technical or logistical measures (e.g., cookies, email confirmation, phone calls) were used to detect/prevent these.

| | 1 | 2 | 3 | 4 | 5 | |
|---|---|---|---|---|---|---|
| subitem not at all important | ○ | ○ | ◉ | ○ | ○ | essential |

Clear selection

Does your paper address subitem 4a-ii? *

Copy and paste relevant sections from the manuscript (include quotes in quotation marks "like this" to indicate direct quotes from your manuscript), or elaborate on this item by providing additional information not in the ms, or briefly explain why the item is not applicable/relevant for your study

"The study was performed remotely on an ambulatory basis...

Participants were recruited in the United States through web media (Facebook and Google Ads). Potential participants were asked to provide contact information and answer questions on demographics (gender, age, employment status, location via city and state, race/ethnicity), smartphone ownership, and smoking attitudes and behavior (Stage of Change and CPD) using an online screening form. Study staff reviewed each online screening form.

Using non-proportional quota sampling, potential participants were called on a first-come-first-served basis, with the aim to enroll 40-60% men, no more than 50% of participants from any decade-spanning age group (e.g. 30-39 years of age.), no more than 70% of participants in the non-Hispanic white race category and up to 20% not employed. The goals of these non-proportional quota sampling ranges were to ensure representation among men, racial/ethnic minorities, age groups, and individuals with varying socioeconomic status. Regarding the non-proportional quota sampling for employment, at the time of protocol design (March and April, 2021) the unemployment rate in the U.S. was 6.0% [16]. Acknowledging a higher unemployment rate among people who smoke [17-20], and the desire to include individuals who either do not receive payment for their work or are not pursuing employment (stay-at-home parents, caretakers, students, retired individuals) we sought to enroll up to 20% of participants who did not have compensated employment.

During the screening phone call, potential participants were asked questions to confirm study eligibility. During this call, study personnel informed the potential participant of the study details and answered any questions.

Potential eligible participants who wanted to proceed with the study were emailed an electronic HIPAA Authorization form and an electronic Informed Consent Form (ICF), which they signed before participating in this study...

...Biovalidation was sought at 12 and 26 weeks (and will be sought at 52 weeks) in individuals who reported 7-day (or greater) PPA on the associated questionnaire. A video call with study staff and the participant was scheduled for within 7 days following the participant’s response to the associated questionnaire."

### 4a-iii) Information giving during recruitment

Information given during recruitment. Specify how participants were briefed for recruitment and in the informed consent procedures (e.g., publish the informed consent documentation as appendix, see also item X26), as this information may have an effect on user self-selection, user expectation and may also bias results.

| | 1 | 2 | 3 | 4 | 5 | |
|---|---|---|---|---|---|---|
| subitem not at all important | ○ | ◉ | ○ | ○ | ○ | essential |

Clear selection

### Does your paper address subitem 4a-iii?

Copy and paste relevant sections from the manuscript (include quotes in quotation marks "like this" to indicate direct quotes from your manuscript), or elaborate on this item by providing additional information not in the ms, or briefly explain why the item is not applicable/relevant for your study

"Participants were recruited in the United States through web media (Facebook and Google Ads). Potential participants were asked to provide contact information and answer questions on demographics (gender, age, employment status, location via city and state, race/ethnicity), smartphone ownership, and smoking attitudes and behavior (Stage of Change and CPD) using an online screening form. Study staff reviewed each online screening form...

...During the screening phone call, potential participants were asked questions to confirm study eligibility. During this call, study personnel informed the potential participant of the study details and answered any questions.

Potential eligible participants who wanted to proceed with the study were emailed an electronic HIPAA Authorization form and an electronic Informed Consent Form (ICF), which they signed before participating in this study..."

Participants were read a standardized script during the screening/information phone call. This script was approved by the IRB. Potential participants were aware of the possible program functions they may or may not have access to during the study: in-app text based coaching, access to an online moderated community forum and a personal carbon monoxide breath sensor. Potential participants were aware all study participants would have access to an app-based cessation program and up to 12 weeks of free nicotine replacement therapy.

4b) Settings and locations where the data were collected

Does your paper address CONSORT subitem 4b? *

Copy and paste relevant sections from the manuscript (include quotes in quotation marks "like this" to indicate direct quotes from your manuscript), or elaborate on this item by providing additional information not in the ms, or briefly explain why the item is not applicable/relevant for your study

"Data were self-reported via weekly online questionnaires for 12 weeks and at 26 weeks... PPA and continuous abstinence were biovalidated via breath CO samples...

Data collection was performed via online questionnaires at baseline, weekly for the first 12 weeks, and at the 26-week follow-up. Collection of participant feedback on one's assigned smoking cessation program was primarily over the first 12 weeks of the study so as to obtain input temporally closest to program use. Study data were imported directly into a secure database (PostgreSQL, PostgreSQL Global Development Group)...

...Biovalidation was sought at 12 and 26 weeks in individuals who reported 7-day (or greater) PPA on the associated questionnaire. A video call with study staff and the participant was scheduled for within 7 days following the participant's response to the associated questionnaire...On the video call, participants held the breath sensor up to the screen immediately after completing the breath sample so that study staff could see and record the CO ppm measurement on the sensor screen. "

4b-i) Report if outcomes were (self-)assessed through online questionnaires

Clearly report if outcomes were (self-)assessed through online questionnaires (as common in web-based trials) or otherwise.

| | 1 | 2 | 3 | 4 | 5 | |
|---|---|---|---|---|---|---|
| subitem not at all important | ○ | ○ | ○ | ◉ | ○ | essential |

Clear selection

Does your paper address subitem 4b-i? *

Copy and paste relevant sections from the manuscript (include quotes in quotation marks "like this" to indicate direct quotes from your manuscript), or elaborate on this item by providing additional information not in the ms, or briefly explain why the item is not applicable/relevant for your study

"Data were self-reported via weekly online questionnaires for 12 weeks and at 26 weeks... PPA and continuous abstinence were biovalidated via breath CO samples...

...Data collection was performed via online questionnaires at baseline, weekly for the first 12 weeks, and at the 26-week follow-up. Collection of participant feedback on one's assigned smoking cessation program was primarily over the first 12 weeks of the study so as to obtain input temporally closest to program use. Study data were imported directly into a secure database (PostgreSQL, PostgreSQL Global Development Group)...

...Biovalidation was sought at 12 and 26 weeks in individuals who reported 7-day (or greater) PPA on the associated questionnaire. A video call with study staff and the participant was scheduled for within 7 days following the participant's response to the associated questionnaire...On the video call, participants held the breath sensor up to the screen immediately after completing the breath sample so that study staff could see and record the CO ppm measurement on the sensor screen."

4b-ii) Report how institutional affiliations are displayed

Report how institutional affiliations are displayed to potential participants [on ehealth media], as affiliations with prestigious hospitals or universities may affect volunteer rates, use, and reactions with regards to an intervention.(Not a required item – describe only if this may bias results)

| | 1 | 2 | 3 | 4 | 5 | |
|---|---|---|---|---|---|---|
| subitem not at all important | ○ | ○ | ◉ | ○ | ○ | essential |

Clear selection

Does your paper address subitem 4b-ii?

Copy and paste relevant sections from the manuscript (include quotes in quotation marks "like this" to indicate direct quotes from your manuscript), or elaborate on this item by providing additional information not in the ms, or briefly explain why the item is not applicable/relevant for your study

Your answer

5) The interventions for each group with sufficient details to allow replication, including how and when they were actually administered

5-i) Mention names, credential, affiliations of the developers, sponsors, and owners

Mention names, credential, affiliations of the developers, sponsors, and owners [6] (if authors/evaluators are owners or developer of the software, this needs to be declared in a “Conflict of interest” section or mentioned elsewhere in the manuscript).

| | 1 | 2 | 3 | 4 | 5 | |
|---|---|---|---|---|---|---|
| subitem not at all important | ○ | ○ | ○ | ◉ | ○ | essential |

Clear selection

Does your paper address subitem 5-i?

Copy and paste relevant sections from the manuscript (include quotes in quotation marks "like this" to indicate direct quotes from your manuscript), or elaborate on this item by providing additional information not in the ms, or briefly explain why the item is not applicable/relevant for your study

"Conflicts of Interest

JM, CF, MU, DB, and DU are current employees of Pivot Health Technologies Inc. (Pivot), the developer of the Pivot smoking cessation program. They receive salary and stock options from Pivot. DU is the President and CEO of Pivot and an investor in the company. JG is a paid statistical consultant."

5-ii) Describe the history/development process

Describe the history/development process of the application and previous formative evaluations (e.g., focus groups, usability testing), as these will have an impact on adoption/use rates and help with interpreting results.

| | 1 | 2 | 3 | 4 | 5 | |
|---|---|---|---|---|---|---|
| subitem not at all important | ○ | ◉ | ○ | ○ | ○ | essential |

Clear selection

Does your paper address subitem 5-ii?

Copy and paste relevant sections from the manuscript (include quotes in quotation marks "like this" to indicate direct quotes from your manuscript), or elaborate on this item by providing additional information not in the ms, or briefly explain why the item is not applicable/relevant for your study

"A cohort study of the Pivot program was published in 2021. During the study, Pivot included a mobile app, personal CO breath sensor, and text-based human coaching. At 3 months post-program completion (mean 7.2 months after enrollment), 32.0% (ITT) and 37.5% (Completer) of participants achieved 7-day PPA; 27.6% (ITT) and 32.4% (Completer) reported 30-day PPA [15]. The Pivot program has since undergone updates, and now includes access to NRT and a moderated online community. These changes, the need for long-term results for app-based cessation programs, and the ongoing need to assess the performance of Pivot within the context of current smoking cessation programs, warrant new investigation of the Pivot program..."

### 5-iii) Revisions and updating

Revisions and updating. Clearly mention the date and/or version number of the application/intervention (and comparator, if applicable) evaluated, or describe whether the intervention underwent major changes during the evaluation process, or whether the development and/or content was “frozen” during the trial. Describe dynamic components such as news feeds or changing content which may have an impact on the replicability of the intervention (for unexpected events see item 3b).

| | 1 | 2 | 3 | 4 | 5 | |
|---|---|---|---|---|---|---|
| subitem not at all important | ○ | ○ | ◉ | ○ | ○ | essential |

Clear selection

### Does your paper address subitem 5-iii?

Copy and paste relevant sections from the manuscript (include quotes in quotation marks "like this" to indicate direct quotes from your manuscript), or elaborate on this item by providing additional information not in the ms, or briefly explain why the item is not applicable/relevant for your study

The intervention and comparator did not undergo major changes during the evaluation process.

### 5-iv) Quality assurance methods

Provide information on quality assurance methods to ensure accuracy and quality of information provided [1], if applicable.

| | 1 | 2 | 3 | 4 | 5 | |
|---|---|---|---|---|---|---|
| subitem not at all important | ○ | ◉ | ○ | ○ | ○ | essential |

Clear selection

Does your paper address subitem 5-iv?

Copy and paste relevant sections from the manuscript (include quotes in quotation marks "like this" to indicate direct quotes from your manuscript), or elaborate on this item by providing additional information not in the ms, or briefly explain why the item is not applicable/relevant for your study

Your answer

5-v) Ensure replicability by publishing the source code, and/or providing screenshots/screen-capture video, and/or providing flowcharts of the algorithms used

Ensure replicability by publishing the source code, and/or providing screenshots/screen-capture video, and/or providing flowcharts of the algorithms used. Replicability (i.e., other researchers should in principle be able to replicate the study) is a hallmark of scientific reporting.

| | 1 | 2 | 3 | 4 | 5 | |
|---|---|---|---|---|---|---|
| subitem not at all important | ○ | ○ | ◉ | ○ | ○ | essential |

Clear selection

Does your paper address subitem 5-v?

Copy and paste relevant sections from the manuscript (include quotes in quotation marks "like this" to indicate direct quotes from your manuscript), or elaborate on this item by providing additional information not in the ms, or briefly explain why the item is not applicable/relevant for your study

Screenshots are provided as a Multi-media appendix

5-vi) Digital preservation

Digital preservation: Provide the URL of the application, but as the intervention is likely to change or disappear over the course of the years; also make sure the intervention is archived (Internet Archive, webcitation.org, and/or publishing the source code or screenshots/videos alongside the article). As pages behind login screens cannot be archived, consider creating demo pages which are accessible without login.

| | 1 | 2 | 3 | 4 | 5 | |
|---|---|---|---|---|---|---|
| subitem not at all important | ○ | ○ | ◉ | ○ | ○ | essential |

Clear selection

Does your paper address subitem 5-vi?

Copy and paste relevant sections from the manuscript (include quotes in quotation marks "like this" to indicate direct quotes from your manuscript), or elaborate on this item by providing additional information not in the ms, or briefly explain why the item is not applicable/relevant for your study

https://apple.com/us/app/pivot-journey-quit-smoking/id1236893700 https://pivot.co/ https://play.google.com/store/apps/details?id=com.carrot.pivot&hl=en_US&gl=US

5-vii) Access

Access: Describe how participants accessed the application, in what setting/context, if they had to pay (or were paid) or not, whether they had to be a member of specific group. If known, describe how participants obtained “access to the platform and Internet” [1]. To ensure access for editors/reviewers/readers, consider to provide a “backdoor” login account or demo mode for reviewers/readers to explore the application (also important for archiving purposes, see vi).

| | 1 | 2 | 3 | 4 | 5 | |
|---|---|---|---|---|---|---|
| subitem not at all important | ○ | ○ | ◉ | ○ | ○ | essential |

Clear selection

Does your paper address subitem 5-vii? *

Copy and paste relevant sections from the manuscript (include quotes in quotation marks "like this" to indicate direct quotes from your manuscript), or elaborate on this item by providing additional information not in the ms, or briefly explain why the item is not applicable/relevant for your study

Participants were emailed a link with log-on code (if applicable) for their respective app to get started. They did not have to pay to access the app. They were not paid to access the app.

5-viii) Mode of delivery, features/functionalities/components of the intervention and comparator, and the theoretical framework

Describe mode of delivery, features/functionalities/components of the intervention and comparator, and the theoretical framework [6] used to design them (instructional strategy [1], behaviour change techniques, persuasive features, etc., see e.g., [7, 8] for terminology). This includes an in-depth description of the content (including where it is coming from and who developed it) [1]," whether [and how] it is tailored to individual circumstances and allows users to track their progress and receive feedback" [6]. This also includes a description of communication delivery channels and – if computer-mediated communication is a component – whether communication was synchronous or asynchronous [6]. It also includes information on presentation strategies [1], including page design principles, average amount of text on pages, presence of hyperlinks to other resources, etc. [1].

| | 1 | 2 | 3 | 4 | 5 | |
|---|---|---|---|---|---|---|
| subitem not at all important | ○ | ○ | ● | ○ | ○ | essential |

Clear selection

Does your paper address subitem 5-viii? *

Copy and paste relevant sections from the manuscript (include quotes in quotation marks "like this" to indicate direct quotes from your manuscript), or elaborate on this item by providing additional information not in the ms, or briefly explain why the item is not applicable/relevant for your study

"Pivot is a 12-month digital smoking cessation program based on the USCPG for tobacco cessation. Pivot includes the Pivot Breath Sensor and Pivot app (Pivot Health Technologies Inc., San Carlos, CA).

The Pivot Breath Sensor is a portable, personal mobile breath sensor that measures the level of carbon monoxide (CO) in exhaled breath. The user submits a breath sample by exhaling into the sensor mouthpiece. The sensor displays the exhaled breath CO value in parts per million (ppm) to the user directly on the device. When paired to the user's smartphone, the user's CO values also populate the Pivot app, where they can be accessed by the user. Displayed CO values are color coded and categorized as most consistent with not smoking (green, 0-6 ppm), possibly smoking (orange, 7-9 ppm) or smoking (red, ≥ 10 ppm). There was no required use of the sensor, however, participants were informed that suggested use of the sensor is four times per day, spread out over the course of the day, acknowledging they should use the sensor as it best fits with their lives. Users may use the sensor to link their smoking behavior and CO values and track their progress in reducing or quitting smoking.

The self-guided Pivot app leverages evidence-based principles and clinical best practices. This includes the USCPG-recommended 5 As (Ask, Advise, Assess, Assist, and Arrange), tailoring on readiness to quit [28], the provision of Food and Drug Administration (FDA)-approved NRT with accompanying education on use and adherence [28-30], the incorporation of effective methods for smoking cessation based on cognitive behavioral therapy (CBT) and self-determination theory [31-33] and CBT-based counseling through a live, dedicated coach [28,32,34]. Pivot app functions include interactive educational activities, the ability to log cigarettes, set a quit date, create a quit plan, complete practice quits (1-24 hours in duration), play educational games, watch educational videos, interact with one's dedicated human coach via in-app text messaging, view CO breath sample values and trends, learn about and then order NRT, access the moderated online Pivot community discussion forum, share goals and progress with the online Pivot community discussion forum or one's social network via text messaging or email, and complete daily check-ins after quit date.

The educational journey in the Pivot App comprises 4 tracts: Learn, Reduce, Prepare to Quit, and Maintain My Quit, and is designed to accommodate smokers along the spectrum of readiness to quit. Participants may choose to focus on building self-awareness and learn more about their smoking behavior, create and practice their plan to quit or reduce smoking, make a quit attempt, focus on staying quit, or any combination thereof. Accordingly, participants may navigate between tracts as desired, to access content most relevant to their goals and needs.

Pivot users are assigned a human coach with whom they work one-on-one over the duration

of their use of Pivot (up to one year). Communication between coach and Pivot user is via asynchronous in-app text messaging. Pivot coaches are tobacco treatment specialists. The coach reaches out periodically, approximately once per week, during the participant's active use of Pivot. Participants may reach out to their coach whenever and however often they like.

Pivot users may access the moderated online discussion community through the Pivot app. The forum is moderated by a tobacco treatment specialist. The online community forum is a place to give and receive support and advice from others going through the Pivot program.

Control: QuitGuide

QuitGuide is a product of Smokefree.gov—a smoking cessation resource created by the Tobacco Control Research Branch at the National Cancer Institute (NCI) in collaboration with tobacco control professionals and smoking cessation experts, and with input from ex-smokers [35]. A well-established smoking cessation app, QuitGuide has been used in previous RCTs in which digital smoking cessation programs were compared [4,5]. The app focuses on helping users understand their smoking patterns and build the skills needed to become and stay smoke-free [35]. Specifically, QuitGuide helps users: focus on motivations to quit; prepare to quit through developing a quit plan, identifying and planning how to address triggers and moods, teaching about FDA-approved smoking cessation medications, and identifying and providing access to social support; quit smoking by acknowledging user progress and teaching skills to address cravings; and stay quit by presenting tips and motivations to stay smoke-free and address slips if they occur.  QuitGuide app functions include educational reading activities, including focus on FDA-approved cessation medications and associated adherence. Additional QuitGuide app functions comprise tracking and reviewing cigarettes, moods, triggers and cravings, setting tip message notifications for locations and times when one is prone to smoke, setting a quit date, creating a quit plan, completing journal entries, sharing goals and progress with one's social network via text messaging or email, accessing additional chat and phone support, and providing updates on quit status after quit date."

### 5-ix) Describe use parameters

Describe use parameters (e.g., intended “doses” and optimal timing for use). Clarify what instructions or recommendations were given to the user, e.g., regarding timing, frequency, heaviness of use, if any, or was the intervention used ad libitum.

| | 1 | 2 | 3 | 4 | 5 | |
|---|---|---|---|---|---|---|
| subitem not at all important | ○ | ○ | ● | ○ | ○ | essential |

Clear selection

### Does your paper address subitem 5-ix?

Copy and paste relevant sections from the manuscript (include quotes in quotation marks "like this" to indicate direct quotes from your manuscript), or elaborate on this item by providing additional information not in the ms, or briefly explain why the item is not applicable/relevant for your study

During the screening call, participants were told that suggested use of the breath sensor (should they receive one) was 4 times a day, spread out over the course of the day, acknowledging they should ultimately use the sensor how it best suits their life. They were also told, "We suggest you use all components of your assigned quit smoking program during the study." No other use recommendations were made.

### 5-x) Clarify the level of human involvement

Clarify the level of human involvement (care providers or health professionals, also technical assistance) in the e-intervention or as co-intervention (detail number and expertise of professionals involved, if any, as well as “type of assistance offered, the timing and frequency of the support, how it is initiated, and the medium by which the assistance is delivered”. It may be necessary to distinguish between the level of human involvement required for the trial, and the level of human involvement required for a routine application outside of a RCT setting (discuss under item 21 – generalizability).

| | 1 | 2 | 3 | 4 | 5 | |
|---|---|---|---|---|---|---|
| subitem not at all important | ○ | ○ | ● | ○ | ○ | essential |

Clear selection

Does your paper address subitem 5-x?

Copy and paste relevant sections from the manuscript (include quotes in quotation marks "like this" to indicate direct quotes from your manuscript), or elaborate on this item by providing additional information not in the ms, or briefly explain why the item is not applicable/relevant for your study

"Pivot users are assigned a human coach with whom they work one-on-one over the duration of their use of Pivot (up to one year). Communication between coach and Pivot user is via asynchronous in-app text messaging. Pivot coaches are tobacco treatment specialists. The coach reaches out periodically, approximately once per week, during the participant's active use of Pivot. Participants may reach out to their coach whenever and however often they like."

5-xi) Report any prompts/reminders used

Report any prompts/reminders used: Clarify if there were prompts (letters, emails, phone calls, SMS) to use the application, what triggered them, frequency etc. It may be necessary to distinguish between the level of prompts/reminders required for the trial, and the level of prompts/reminders for a routine application outside of a RCT setting (discuss under item 21 – generalizability).

| | 1 | 2 | 3 | 4 | 5 | |
|---|---|---|---|---|---|---|
| subitem not at all important | ○ | ○ | ◉ | ○ | ○ | essential |

Clear selection

Does your paper address subitem 5-xi? *

Copy and paste relevant sections from the manuscript (include quotes in quotation marks "like this" to indicate direct quotes from your manuscript), or elaborate on this item by providing additional information not in the ms, or briefly explain why the item is not applicable/relevant for your study

"A total of 6 reminders to prompt use of the program were emailed to all participants every other week over the first 12 weeks of the study"

5-xii) Describe any co-interventions (incl. training/support)

Describe any co-interventions (incl. training/support): Clearly state any interventions that are provided in addition to the targeted eHealth intervention, as ehealth intervention may not be designed as stand-alone intervention. This includes training sessions and support [1]. It may be necessary to distinguish between the level of training required for the trial, and the level of training for a routine application outside of a RCT setting (discuss under item 21 – generalizability.

| | 1 | 2 | 3 | 4 | 5 | |
|---|---|---|---|---|---|---|
| subitem not at all important | ○ | ○ | ◉ | ○ | ○ | essential |

Clear selection

Does your paper address subitem 5-xii? *

Copy and paste relevant sections from the manuscript (include quotes in quotation marks "like this" to indicate direct quotes from your manuscript), or elaborate on this item by providing additional information not in the ms, or briefly explain why the item is not applicable/relevant for your study

No additional training/interventions were required of study participants. The eHealth interventions (Pivot and QuitGuide) were designed as stand-alone interventions. Study training staff trained on how to conduct the biovalidation visits, which were videocalls during which they read a standardized script to participants.

6a) Completely defined pre-specified primary and secondary outcome measures, including how and when they were assessed

Does your paper address CONSORT subitem 6a? *

Copy and paste relevant sections from the manuscript (include quotes in quotation marks "like this" to indicate direct quotes from your manuscript), or elaborate on this item by providing additional information not in the ms, or briefly explain why the item is not applicable/relevant for your study

"Study outcomes focused on four areas: user engagement and retention, attitudes toward quitting, smoking behavior, and participant feedback.

User engagement and retention
The pre-registered primary outcome of the study was total app openings in Pivot vs QuitGuide at 12 weeks. Additional outcomes included the number of days and number of weeks with ≥ 1 app opening. App openings were self-reported weekly for the first 12 weeks of the study. Self-report of app utilization has been reported previously [5] and was necessary because automatic recording of this information was not enabled for QuitGuide...

...Data collection:
Data collection was performed via online questionnaires at baseline, weekly for the first 12 weeks, and at the 26-week follow-up. Collection of participant feedback on one's assigned smoking cessation program was primarily over the first 12 weeks of the study so as to obtain input temporally closest to program use."

6a-i) Online questionnaires: describe if they were validated for online use and apply CHERRIES items to describe how the questionnaires were designed/deployed

If outcomes were obtained through online questionnaires, describe if they were validated for online use and apply CHERRIES items to describe how the questionnaires were designed/deployed [9].

| | 1 | 2 | 3 | 4 | 5 | |
|---|---|---|---|---|---|---|
| subitem not at all important | ○ | ◉ | ○ | ○ | ○ | essential |

Clear selection

Does your paper address subitem 6a-i?

Copy and paste relevant sections from manuscript text

Your answer

6a-ii) Describe whether and how “use” (including intensity of use/dosage) was defined/measured/monitored

Describe whether and how “use” (including intensity of use/dosage) was defined/measured/monitored (logins, logfile analysis, etc.). Use/adoption metrics are important process outcomes that should be reported in any ehealth trial.

| | 1 | 2 | 3 | 4 | 5 | |
|---|---|---|---|---|---|---|
| subitem not at all important | ○ | ○ | ◉ | ○ | ○ | essential |

Clear selection

Does your paper address subitem 6a-ii?

Copy and paste relevant sections from manuscript text

Participants were asked the following questions weekly for the first 12 weeks of the study:
1. Approximately how many days did you open your study app over the last 7 days? Select one: ▫ 0 ▫ 1 ▫ 2 ▫ 3 ▫ 4 ▫ 5 ▫ 6 ▫ 7
2. Approximately how many times have you opened your study app over the last 7 days?

6a-iii) Describe whether, how, and when qualitative feedback from participants was obtained

Describe whether, how, and when qualitative feedback from participants was obtained (e.g., through emails, feedback forms, interviews, focus groups).

| | 1 | 2 | 3 | 4 | 5 | |
|---|---|---|---|---|---|---|
| subitem not at all important | ○ | ◉ | ○ | ○ | ○ | essential |

Clear selection

Does your paper address subitem 6a-iii?

Copy and paste relevant sections from manuscript text

Qualitative feedback was obtained from the online questionnaires participants completed. Examples of qualitative questions asked include, 'How would you improve the experience of your assigned program?' 'What has been the most helpful part of your program?' 'What has been the most frustrating part of your program?' Responses to these questions will be addressed elsewhere, as they are outside the scope of the current paper.

6b) Any changes to trial outcomes after the trial commenced, with reasons

Does your paper address CONSORT subitem 6b? *

Copy and paste relevant sections from the manuscript (include quotes in quotation marks "like this" to indicate direct quotes from your manuscript), or elaborate on this item by providing additional information not in the ms, or briefly explain why the item is not applicable/relevant for your study

There were no changes to trial outcomes after the trial commenced.

7a) How sample size was determined

NPT: When applicable, details of whether and how the clustering by care provides or centers was addressed

7a-i) Describe whether and how expected attrition was taken into account when calculating the sample size

Describe whether and how expected attrition was taken into account when calculating the sample size.

| | 1 | 2 | 3 | 4 | 5 | |
|---|---|---|---|---|---|---|
| subitem not at all important | ○ | ○ | ● | ○ | ○ | essential |

Clear selection

Does your paper address subitem 7a-i?

Copy and paste relevant sections from manuscript title (include quotes in quotation marks "like this" to indicate direct quotes from your manuscript), or elaborate on this item by providing additional information not in the ms, or briefly explain why the item is not applicable/relevant for your study

"As this is a pilot RCT and the first assessment of Pivot compared to usual care, the sample size is powered to show differences in engagement, specifically, the number of times participants opened their assigned app over the first 12 weeks of the study. In previous clinical studies, Pivot mean app openings were 24.2-38.7 (SD range 20.8-25.9) by 90 days (data on file). In addition, Bricker et al. reported app openings comparing ACT-based smoking cessation apps (SmartQuit and iCanQuit) to QuitGuide. In one study, at 2-month follow-up, Bricker et al. reported mean (SD) app openings were 37.2 (46.1) for SmartQuit and 15.2 (13.6) for QuitGuide [5]. In a subsequent study, at 12-month follow-up, mean (SD) app openings were 37.5 (88.4) for iCanQuit and 9.9 (50.0) for QuitGuide [4].

Based on this data, we estimated mean (SD) 25 (25) app openings in the Pivot intervention arm vs. 15 (19) app openings in the QuitGuide control arm at 12 weeks. Detecting a difference of 10 app openings between Pivot and QuitGuide with 0.8 power and 0.05 alpha would require 156.7 participants, which we round up to 158. In a previous study, 272/319 (85.3%) participants completed the end-of-Pivot questionnaire at a mean (SD) 4.1 (1.4) months after enrollment [3743]. In assessing the primary endpoint at 3 months (12 weeks), we included an expected 15% attrition rate, with the aim to enroll up to 180 participants (up to 90 in each arm)."

7b) When applicable, explanation of any interim analyses and stopping guidelines

Does your paper address CONSORT subitem 7b? *

Copy and paste relevant sections from the manuscript (include quotes in quotation marks "like this" to indicate direct quotes from your manuscript), or elaborate on this item by providing additional information not in the ms, or briefly explain why the item is not applicable/relevant for your study

Explanation of interim analyses and stopping guidelines is not applicable.

8a) Method used to generate the random allocation sequence

NPT: When applicable, how care providers were allocated to each trial group

Does your paper address CONSORT subitem 8a? *

Copy and paste relevant sections from the manuscript (include quotes in quotation marks "like this" to indicate direct quotes from your manuscript), or elaborate on this item by providing additional information not in the ms, or briefly explain why the item is not applicable/relevant for your study

"The allocation sequence was provided by Study Randomizer software application (2017) [21]."

8b) Type of randomisation; details of any restriction (such as blocking and block size)

Does your paper address CONSORT subitem 8b? *

Copy and paste relevant sections from the manuscript (include quotes in quotation marks "like this" to indicate direct quotes from your manuscript), or elaborate on this item by providing additional information not in the ms, or briefly explain why the item is not applicable/relevant for your study

"Participants were randomly assigned in a computer-generated 1:1 ratio to either QuitGuide or Pivot using randomly permuted blocks of size 2 and 4."

9) Mechanism used to implement the random allocation sequence (such as sequentially numbered containers), describing any steps taken to conceal the sequence until interventions were assigned

Does your paper address CONSORT subitem 9? *

Copy and paste relevant sections from the manuscript (include quotes in quotation marks "like this" to indicate direct quotes from your manuscript), or elaborate on this item by providing additional information not in the ms, or briefly explain why the item is not applicable/relevant for your study

"The allocation sequence was provided by Study Randomizer software application (2017) [21]...Researchers were blinded to treatment allocation until after randomization was performed."

10) Who generated the random allocation sequence, who enrolled participants, and who assigned participants to interventions

Does your paper address CONSORT subitem 10? *

Copy and paste relevant sections from the manuscript (include quotes in quotation marks "like this" to indicate direct quotes from your manuscript), or elaborate on this item by providing additional information not in the ms, or briefly explain why the item is not applicable/relevant for your study

The Study Randomizer software application generated the random allocation sequence and assigned participants to interventions. Participants were considered enrolled after completing the following 3 tasks: electronically signing the informed consent form, completing the Baseline Questionnaire and reporting the date they first logged in to their assigned app. Study personnel designated participants as enrolled after they completed these 3 criteria.

11a) If done, who was blinded after assignment to interventions (for example, participants, care providers, those assessing outcomes) and how

NPT: Whether or not administering co-interventions were blinded to group assignment

11a-i) Specify who was blinded, and who wasn't

Specify who was blinded, and who wasn't. Usually, in web-based trials it is not possible to blind the participants [1, 3] (this should be clearly acknowledged), but it may be possible to blind outcome assessors, those doing data analysis or those administering co-interventions (if any).

| | 1 | 2 | 3 | 4 | 5 | |
|---|---|---|---|---|---|---|
| subitem not at all important | ○ | ○ | ● | ○ | ○ | essential |

Clear selection

Does your paper address subitem 11a-i? *

Copy and paste relevant sections from the manuscript (include quotes in quotation marks "like this" to indicate direct quotes from your manuscript), or elaborate on this item by providing additional information not in the ms, or briefly explain why the item is not applicable/relevant for your study

"Researchers were blinded to treatment allocation until after randomization was performed." As it was not possible in this web-based trial, participants were not blinded to treatment allocation.

11a-ii) Discuss e.g., whether participants knew which intervention was the "intervention of interest" and which one was the "comparator"

Informed consent procedures (4a-ii) can create biases and certain expectations - discuss e.g., whether participants knew which intervention was the "intervention of interest" and which one was the "comparator".

| | 1 | 2 | 3 | 4 | 5 | |
|---|---|---|---|---|---|---|
| subitem not at all important | ○ | ○ | ● | ○ | ○ | essential |

Clear selection

### Does your paper address subitem 11a-ii?

Copy and paste relevant sections from the manuscript (include quotes in quotation marks "like this" to indicate direct quotes from your manuscript), or elaborate on this item by providing additional information not in the ms, or briefly explain why the item is not applicable/relevant for your study

During the informed consent process, participants were informed of possible program features they may or may not have, such as a carbon monoxide breath sensor, app-based human coaching, and access to a moderated online community forum. Participants were not informed of specific characteristics of the two programs (for example, that one program was Pivot and comprised features A-E, and the other program was QuitGuide and comprised features A-B).

### 11b) If relevant, description of the similarity of interventions

(this item is usually not relevant for ehealth trials as it refers to similarity of a placebo or sham intervention to a active medication/intervention)

### Does your paper address CONSORT subitem 11b? *

Copy and paste relevant sections from the manuscript (include quotes in quotation marks "like this" to indicate direct quotes from your manuscript), or elaborate on this item by providing additional information not in the ms, or briefly explain why the item is not applicable/relevant for your study

The similarity of interventions is addressed in the manuscript text included in 5-viii above.

### 12a) Statistical methods used to compare groups for primary and secondary outcomes

NPT: When applicable, details of whether and how the clustering by care providers or centers was addressed

Does your paper address CONSORT subitem 12a? *

Copy and paste relevant sections from the manuscript (include quotes in quotation marks "like this" to indicate direct quotes from your manuscript), or elaborate on this item by providing additional information not in the ms, or briefly explain why the item is not applicable/relevant for your study

"In this pilot RCT, differences between the Pivot intervention arm and the QuitGuide control arm were evaluated. Baseline comparisons and changes from baseline used unadjusted statistical tests. For numerical data we calculated the mean (SD) and used a t-test. For categorical data, we calculated the proportions and used the Chi-square test or Fisher's exact test. For results where a change from baseline can be measured, each participant's baseline data served as their control to calculate a difference with a latter timepoint (e.g. CPD, SASEQ, STQ, DTQ), which then served as the measurement and a paired t-test was used to test for a difference from zero.

For outcomes, regression analyses were adjusted for the randomization stratification covariates to detect differences between the treatment and control arms. Linear regression was used for numerical data to obtain a point estimate of the mean difference. For count outcomes, the IRR was estimated using Poisson regression when the variance to mean ratio was close to one, or negative binomial regressions when the variance to mean ratio was greater than one. For binary outcomes, the OR was estimated using logistic regression, and the relative risk estimated using either log-link binomial regression or log-link Poisson regression with robust estimators [44]. For binary outcomes where there was a very high frequency response (e.g. ≥ 95%), only the relative risk is presented. For multicategory outcomes of three or more, multinomial logistic regression was used to test for proportion differences between the arms. If the multinomial logistic regression model did not converge, categories were collapsed. Statistical significance was set at P<.05. Analyses were conducted using SAS version 9.4 (SAS Institute, Cary, NC).
In the assessment of quit rates (self-reported and biovalidated PPA and continuous abstinence, and self-reported abstinence from all tobacco products), two sets of analyses were performed. In the ITT analysis, individuals who did not respond to PPA questions were assumed to be smoking. A study responder analysis was also performed, which only included individuals who completed the questionnaire from the associated timepoint. For the outcomes of quit attempts and the proportion who reduced CPD by at least 50%, a study completer analysis was performed."

12a-i) Imputation techniques to deal with attrition / missing values

Imputation techniques to deal with attrition / missing values: Not all participants will use the intervention/comparator as intended and attrition is typically high in ehealth trials. Specify how participants who did not use the application or dropped out from the trial were treated in the statistical analysis (a complete case analysis is strongly discouraged, and simple imputation techniques such as LOCF may also be problematic [4]).

| | 1 | 2 | 3 | 4 | 5 | |
|---|---|---|---|---|---|---|
| subitem not at all important | ○ | ○ | ◉ | ○ | ○ | essential |

Clear selection

Does your paper address subitem 12a-i? *

Copy and paste relevant sections from the manuscript (include quotes in quotation marks "like this" to indicate direct quotes from your manuscript), or elaborate on this item by providing additional information not in the ms, or briefly explain why the item is not applicable/relevant for your study

"Survey completion was high at 12 weeks: 97.3% (91/94) in QuitGuide and 97.9% (92/94) in Pivot, and at 6 months: 95.7 % in both QuitGuide ((90/94) and Pivot ((90/94). For this reason, completer and intention to treat analyses were considered appropriate at the 12- and 26-week timepoints.

The primary endpoint of the total number of app openings through 12 weeks was calculated by summing the number of weekly app openings, which were reported by participants weekly and represented total app openings over the preceding 7 days. There were 170 participants who completed all 12 surveys. There were four participants (2 in Pivot and 2 in QuitGuide) who withdrew consent by week three, accounting for 41 incomplete surveys. App openings for these participants were set to zero as they were not participating in the study. This left 14 participants (8 Pivot, 6 QuitGuide) with one or more surveys not completed for a total of 44 incomplete surveys. While this only represented 7.4% of total participants and 2.0% of total surveys, imputation was necessary to calculate the total app openings, total days with app openings and total weeks with app openings.

There was no pattern of missingness upon visual inspection and multiple imputation method was performed using SAS MI Procedure full conditional specification predicted mean matching with 25 imputations [45]. The primary endpoint of total app openings by the intervention and control arms was then compared in a negative binomial regression model adjusted for the four randomization covariates in each of the imputations with SAS MIANALYZE. Similarly, total days with app openings and total weeks with app openings were analyzed using negative binomial regression and Poisson regression, respectively. The mean of the imputed data was used for reporting descriptive statistics."

12b) Methods for additional analyses, such as subgroup analyses and adjusted analyses

Does your paper address CONSORT subitem 12b? *

Copy and paste relevant sections from the manuscript (include quotes in quotation marks "like this" to indicate direct quotes from your manuscript), or elaborate on this item by providing additional information not in the ms, or briefly explain why the item is not applicable/relevant for your study

For subgroup analyses and adjusted analyses, see response to item 12a

X26) REB/IRB Approval and Ethical Considerations [recommended as subheading under "Methods"] (not a CONSORT item)

X26-i) Comment on ethics committee approval

| | 1 | 2 | 3 | 4 | 5 | |
|---|---|---|---|---|---|---|
| subitem not at all important | ○ | ○ | ○ | ◉ | ○ | essential |

Clear selection

Does your paper address subitem X26-i?

Copy and paste relevant sections from the manuscript (include quotes in quotation marks "like this" to indicate direct quotes from your manuscript), or elaborate on this item by providing additional information not in the ms, or briefly explain why the item is not applicable/relevant for your study

"The study was reviewed and approved by the Solutions IRB, LLC (Yarnell, AZ, USA) protocol number 2021/04/38..."

x26-ii) Outline informed consent procedures

Outline informed consent procedures e.g., if consent was obtained offline or online (how? Checkbox, etc.?), and what information was provided (see 4a-ii). See [6] for some items to be included in informed consent documents.

| | 1 | 2 | 3 | 4 | 5 | |
|---|---|---|---|---|---|---|
| subitem not at all important | ○ | ○ | ● | ○ | ○ | essential |

Clear selection

Does your paper address subitem X26-ii?

Copy and paste relevant sections from the manuscript (include quotes in quotation marks "like this" to indicate direct quotes from your manuscript), or elaborate on this item by providing additional information not in the ms, or briefly explain why the item is not applicable/relevant for your study

"Potential eligible participants who wanted to proceed with the study were emailed an electronic HIPAA Authorization form and an electronic Informed Consent Form, which they signed before participating in this study."

X26-iii) Safety and security procedures

Safety and security procedures, incl. privacy considerations, and any steps taken to reduce the likelihood or detection of harm (e.g., education and training, availability of a hotline)

| | 1 | 2 | 3 | 4 | 5 | |
|---|---|---|---|---|---|---|
| subitem not at all important | ○ | ○ | ● | ○ | ○ | essential |

Clear selection

Does your paper address subitem X26-iii?

Copy and paste relevant sections from the manuscript (include quotes in quotation marks "like this" to indicate direct quotes from your manuscript), or elaborate on this item by providing additional information not in the ms, or briefly explain why the item is not applicable/relevant for your study

Study team members had documentation of HIPAA / HITECH Security Awareness training and NIH Protecting Human Research Participants training. The study team takes careful measures to prevent a data breach including use of encryptions, secure connections, and limited access to data. The study team uses appropriate safeguards and complies, where applicable, with 45 C.F.R. Part 164, Subpart C with respect to Electronic Protected Health Information, to prevent use or disclosure of Protected Health Information. Specifically, Pivot Health Technologies Inc. has implemented the administrative, physical, and technical safeguards set forth in 45 C.F.R. §§ 164.308, 164.310, and 164.312 that reasonably and appropriately protect the confidentiality, integrity, and availability of any Protected Health Information that it creates, receives, maintains, or transmits. The QuitGuide app is maintained by the U.S. Government. It is protected by various provisions of Title 18, U.S. Code. The QuitGuide app notes, “NCI complies with requirements for privacy and security established by the Office of Management and Budget (OMB), Department of Health and Human Services (DHHS), and the National Institutes of Health (NIH)."

RESULTS

13a) For each group, the numbers of participants who were randomly assigned, received intended treatment, and were analysed for the primary outcome

NPT: The number of care providers or centers performing the intervention in each group and the number of patients treated by each care provider in each center

Does your paper address CONSORT subitem 13a? *

Copy and paste relevant sections from the manuscript (include quotes in quotation marks "like this" to indicate direct quotes from your manuscript), or elaborate on this item by providing additional information not in the ms, or briefly explain why the item is not applicable/relevant for your study

"From June to October 2021, 3042 online screening forms were received; 533 met the screening eligibility criteria and responded to an initial outbound phone call from study staff. Two hundred and ninety-two of these individuals did not proceed further, most commonly due to ineligibility after the phone call (n=134), or lack of response to subsequent outreach after initial contact (n=111). One hundred and eighty-eight individuals were randomized and completed enrollment (94 in each arm), comprising the ITT sample."

13b) For each group, losses and exclusions after randomisation, together with reasons

Does your paper address CONSORT subitem 13b? (NOTE: Preferably, this is shown in a CONSORT flow diagram) *

Copy and paste relevant sections from the manuscript (include quotes in quotation marks "like this" to indicate direct quotes from your manuscript), or elaborate on this item by providing additional information not in the ms, or briefly explain why the item is not applicable/relevant for your study

This is addressed in the CONSORT flow diagram, which is "Figure 1 Study participant flow: CONSORT diagram" in the manuscript.

13b-i) Attrition diagram

Strongly recommended: An attrition diagram (e.g., proportion of participants still logging in or using the intervention/comparator in each group plotted over time, similar to a survival curve) or other figures or tables demonstrating usage/dose/engagement.

| | 1 | 2 | 3 | 4 | 5 | |
|---|---|---|---|---|---|---|
| subitem not at all important | ○ | ○ | ◉ | ○ | ○ | essential |

Clear selection

Does your paper address subitem 13b-i?

Copy and paste relevant sections from the manuscript or cite the figure number if applicable (include quotes in quotation marks "like this" to indicate direct quotes from your manuscript), or elaborate on this item by providing additional information not in the ms, or briefly explain why the item is not applicable/relevant for your study

We elected to not include an attrition diagram and instead included the following text, "Self-report of logging into their app at least once a week was reported in ≥ 85% of participants in each arm for each week through 12 weeks; in QuitGuide it ranged from 85% to 97% and in Pivot it was 86% to 98%."

14a) Dates defining the periods of recruitment and follow-up

Does your paper address CONSORT subitem 14a? *

Copy and paste relevant sections from the manuscript (include quotes in quotation marks "like this" to indicate direct quotes from your manuscript), or elaborate on this item by providing additional information not in the ms, or briefly explain why the item is not applicable/relevant for your study

"From June to October 2021, 3042 online screening forms were received; 533 met the screening eligibility criteria and responded to an initial outbound phone call from study staff." The last 6-month questionnaire was completed April 28, 2022.

14a-i) Indicate if critical “secular events” fell into the study period

Indicate if critical “secular events” fell into the study period, e.g., significant changes in Internet resources available or “changes in computer hardware or Internet delivery resources”

| | 1 | 2 | 3 | 4 | 5 | |
|---|---|---|---|---|---|---|
| subitem not at all important | ○ | ◉ | ○ | ○ | ○ | essential |

Clear selection

Does your paper address subitem 14a-i?

Copy and paste relevant sections from the manuscript (include quotes in quotation marks "like this" to indicate direct quotes from your manuscript), or elaborate on this item by providing additional information not in the ms, or briefly explain why the item is not applicable/relevant for your study

No critical secular events fell into the study period

14b) Why the trial ended or was stopped (early)

Does your paper address CONSORT subitem 14b? *

Copy and paste relevant sections from the manuscript (include quotes in quotation marks "like this" to indicate direct quotes from your manuscript), or elaborate on this item by providing additional information not in the ms, or briefly explain why the item is not applicable/relevant for your study

6-month data collection is complete. This is a two-year study and is ongoing. The trial has not ended and was not stopped early.

15) A table showing baseline demographic and clinical characteristics for each group
NPT: When applicable, a description of care providers (case volume, qualification, expertise, etc.) and centers (volume) in each group

Does your paper address CONSORT subitem 15? *

Copy and paste relevant sections from the manuscript (include quotes in quotation marks "like this" to indicate direct quotes from your manuscript), or elaborate on this item by providing additional information not in the ms, or briefly explain why the item is not applicable/relevant for your study

See "Table 1. Participant baseline data (n=188)"

15-i) Report demographics associated with digital divide issues

In ehealth trials it is particularly important to report demographics associated with digital divide issues, such as age, education, gender, social-economic status, computer/Internet/ehealth literacy of the participants, if known.

| | 1 | 2 | 3 | 4 | 5 | |
|---|---|---|---|---|---|---|
| subitem not at all important | ○ | ◉ | ○ | ○ | ○ | essential |

Clear selection

Does your paper address subitem 15-i? *

Copy and paste relevant sections from the manuscript (include quotes in quotation marks "like this" to indicate direct quotes from your manuscript), or elaborate on this item by providing additional information not in the ms, or briefly explain why the item is not applicable/relevant for your study

Yes demographics associated with digital divide issues are reported; see "Table 1. Participant baseline data (n=188)"

16) For each group, number of participants (denominator) included in each analysis and whether the analysis was by original assigned groups

16-i) Report multiple "denominators" and provide definitions

Report multiple "denominators" and provide definitions: Report N's (and effect sizes) "across a range of study participation [and use] thresholds" [1], e.g., N exposed, N consented, N used more than x times, N used more than y weeks, N participants "used" the intervention/comparator at specific pre-defined time points of interest (in absolute and relative numbers per group). Always clearly define "use" of the intervention.

| | 1 | 2 | 3 | 4 | 5 | |
|---|---|---|---|---|---|---|
| subitem not at all important | ○ | ○ | ○ | ◉ | ○ | essential |

Clear selection

Does your paper address subitem 16-i? *

Copy and paste relevant sections from the manuscript (include quotes in quotation marks "like this" to indicate direct quotes from your manuscript), or elaborate on this item by providing additional information not in the ms, or briefly explain why the item is not applicable/relevant for your study

Yes all results and tables include denominators across a range of study participation and thresholds, noting that attrition was low, so denominators for enrolled and completer analyses were close.

16-ii) Primary analysis should be intent-to-treat

Primary analysis should be intent-to-treat, secondary analyses could include comparing only “users”, with the appropriate caveats that this is no longer a randomized sample (see 18-i).

| | 1 | 2 | 3 | 4 | 5 | |
|---|---|---|---|---|---|---|
| subitem not at all important | ○ | ○ | ○ | ◉ | ○ | essential |

Clear selection

Does your paper address subitem 16-ii?

Copy and paste relevant sections from the manuscript (include quotes in quotation marks "like this" to indicate direct quotes from your manuscript), or elaborate on this item by providing additional information not in the ms, or briefly explain why the item is not applicable/relevant for your study

Your answer

17a) For each primary and secondary outcome, results for each group, and the estimated effect size and its precision (such as 95% confidence interval)

Does your paper address CONSORT subitem 17a? *

Copy and paste relevant sections from the manuscript (include quotes in quotation marks "like this" to indicate direct quotes from your manuscript), or elaborate on this item by providing additional information not in the ms, or briefly explain why the item is not applicable/relevant for your study

Effects sizes (odds ratios, relative risk, point estimates, incidence rate ratios) and confidence intervals are reported for each group as applicable. See Tables 2 and 3 and the following sections in the Results: User Engagement and Retention, Quit Attempts, Changes in CPD, Cessation Rates, Use of NRT...

17a-i) Presentation of process outcomes such as metrics of use and intensity of use

In addition to primary/secondary (clinical) outcomes, the presentation of process outcomes such as metrics of use and intensity of use (dose, exposure) and their operational definitions is critical. This does not only refer to metrics of attrition (13-b) (often a binary variable), but also to more continuous exposure metrics such as “average session length”. These must be accompanied by a technical description how a metric like a “session” is defined (e.g., timeout after idle time) [1] (report under item 6a).

| | 1 | 2 | 3 | 4 | 5 | |
|---|---|---|---|---|---|---|
| subitem not at all important | ○ | ○ | ◉ | ○ | ○ | essential |

Clear selection

Does your paper address subitem 17a-i?

Copy and paste relevant sections from the manuscript (include quotes in quotation marks "like this" to indicate direct quotes from your manuscript), or elaborate on this item by providing additional information not in the ms, or briefly explain why the item is not applicable/relevant for your study

Our metrics of use included self-reported app openings, days with app openings and weeks with app opening through week 12. Because we did not have access to the QuitGuide use analytic data, we were unable to assess continuous exposure metrics such as average session length.

17b) For binary outcomes, presentation of both absolute and relative effect sizes is recommended

Does your paper address CONSORT subitem 17b? *

Copy and paste relevant sections from the manuscript (include quotes in quotation marks "like this" to indicate direct quotes from your manuscript), or elaborate on this item by providing additional information not in the ms, or briefly explain why the item is not applicable/relevant for your study

Adjusted relative risk was added in addition to the adjusted odds ratio for all binary outcomes. Absolute difference in risk, albeit unadjusted, can be assessed easily from the proportions presented in "Table 3 Smoking cessation rates at 12 and 26 weeks" and we decided not to add this to the table.

18) Results of any other analyses performed, including subgroup analyses and adjusted analyses, distinguishing pre-specified from exploratory

Does your paper address CONSORT subitem 18? *

Copy and paste relevant sections from the manuscript (include quotes in quotation marks "like this" to indicate direct quotes from your manuscript), or elaborate on this item by providing additional information not in the ms, or briefly explain why the item is not applicable/relevant for your study

"Baseline comparisons and changes from baseline used unadjusted statistical tests...For outcomes, regression analyses were adjusted for the randomization stratification covariates to detect differences between the treatment..." "Among participants who did not report 7-day (or greater) PPA at 26 weeks (n=121), CPD were reduced by 44.4% (SD 33.7) from baseline. Within each group, the reduction in CPD from baseline to 26 weeks was significant (P<.001 for both). The reduction in CPD was similar between the two groups: Pivot -39.1% (SD 34.7) vs. QuitGuide -48.9% (SD 32.5) (point estimate 11.6; 95% CI, -0.4, 23.6; P=.06)." "Focusing on participants who did not report 7-day (or greater) PPA at 26 weeks (n=121), the proportion who reduced CPD by ≥50% was similar between the two groups: Pivot 39.3% (22/56) vs. QuitGuide 52.3% (34/65), (OR, 0.5; 95% CI, 0.2, 1.1; P=.10 ; RR, 0.7; 95% CI, 0.5, 1.1; P=.09)."

18-i) Subgroup analysis of comparing only users

A subgroup analysis of comparing only users is not uncommon in ehealth trials, but if done, it must be stressed that this is a self-selected sample and no longer an unbiased sample from a randomized trial (see 16-iii).

| | 1 | 2 | 3 | 4 | 5 | |
|---|---|---|---|---|---|---|
| subitem not at all important | ○ | ○ | ● | ○ | ○ | essential |

Clear selection

Does your paper address subitem 18-i?

Copy and paste relevant sections from the manuscript (include quotes in quotation marks "like this" to indicate direct quotes from your manuscript), or elaborate on this item by providing additional information not in the ms, or briefly explain why the item is not applicable/relevant for your study

We did not do a subgroup analysis comparing only users.

19) All important harms or unintended effects in each group

(for specific guidance see CONSORT for harms)

Does your paper address CONSORT subitem 19? *

Copy and paste relevant sections from the manuscript (include quotes in quotation marks "like this" to indicate direct quotes from your manuscript), or elaborate on this item by providing additional information not in the ms, or briefly explain why the item is not applicable/relevant for your study

There were no important harms or unintended effects in either group.

19-i) Include privacy breaches, technical problems

Include privacy breaches, technical problems. This does not only include physical "harm" to participants, but also incidents such as perceived or real privacy breaches [1], technical problems, and other unexpected/unintended incidents. "Unintended effects" also includes unintended positive effects [2].

| | 1 | 2 | 3 | 4 | 5 | |
|---|---|---|---|---|---|---|
| subitem not at all important | ○ | ○ | ◉ | ○ | ○ | essential |

Clear selection

Does your paper address subitem 19-i?

Copy and paste relevant sections from the manuscript (include quotes in quotation marks "like this" to indicate direct quotes from your manuscript), or elaborate on this item by providing additional information not in the ms, or briefly explain why the item is not applicable/relevant for your study

There were no privacy breaches or technical problems, including incidents such as perceived or real privacy breaches or other unexpected/unintended incidents.

19-ii) Include qualitative feedback from participants or observations from staff/researchers

Include qualitative feedback from participants or observations from staff/researchers, if available, on strengths and shortcomings of the application, especially if they point to unintended/unexpected effects or uses. This includes (if available) reasons for why people did or did not use the application as intended by the developers.

| | 1 | 2 | 3 | 4 | 5 | |
|---|---|---|---|---|---|---|
| subitem not at all important | ○ | ○ | ◉ | ○ | ○ | essential |

Clear selection

Does your paper address subitem 19-ii?

Copy and paste relevant sections from the manuscript (include quotes in quotation marks "like this" to indicate direct quotes from your manuscript), or elaborate on this item by providing additional information not in the ms, or briefly explain why the item is not applicable/relevant for your study

While descriptive categorical data was collected from participants and is included in the manuscript, qualitative data is outside the scope of the current manuscript. See 6a-iii response.

## DISCUSSION

22) Interpretation consistent with results, balancing benefits and harms, and considering other relevant evidence

NPT: In addition, take into account the choice of the comparator, lack of or partial blinding, and unequal expertise of care providers or centers in each group

22-i) Restate study questions and summarize the answers suggested by the data, starting with primary outcomes and process outcomes (use)

Restate study questions and summarize the answers suggested by the data, starting with primary outcomes and process outcomes (use).

| | 1 | 2 | 3 | 4 | 5 | |
|---|---|---|---|---|---|---|
| subitem not at all important | ○ | ○ | ○ | ◉ | ○ | essential |

Clear selection

Does your paper address subitem 22-i? *

Copy and paste relevant sections from the manuscript (include quotes in quotation marks "like this" to indicate direct quotes from your manuscript), or elaborate on this item by providing additional information not in the ms, or briefly explain why the item is not applicable/relevant for your study

"This pilot RCT compared user engagement and retention, change in attitudes towards quitting smoking, change in smoking behavior, and participant feedback in adult smokers randomized to either the Pivot or QuitGuide app-based smoking cessation programs. Program engagement as assessed by total app openings through 12 weeks, the pre-registered primary outcome of the study, was significantly higher in Pivot than in QuitGuide (P<.001). Measures assessing attitudes toward quitting smoking, including self-efficacy (SASEQ), expected success in quitting (STQ) and expected difficulty staying quit (DTQ) improved significantly in each group through 12 weeks, but were not different between groups. Most participants (>96%) made at least one quit attempt, with QuitGuide participants reporting more quit attempts through 26 weeks (P=.003). The study was not powered for differences in quit rates; while self-reported 7- and 30-day quit rates were approximately 10 percentage points higher in Pivot at 26 weeks (e.g. 7-day PPA at 26 weeks was 36.2% in Pivot and 26.6% in QuitGuide, ITT) these differences were not statistically significant. However, differences in biovalidated quit rates were significant at 12 weeks (28.7% Pivot vs. 12.8% QuitGuide, ITT, P=.008) and 26 weeks (27.7% Pivot vs. 14.9%, ITT, P=.03), as was the difference in the biovalidated continuous quit rate at 26 weeks (21.3% Pivot vs. 9.6% QuitGuide, ITT, P=.03). In general, participants rated the Pivot program more favorably, including the set up and impact of the program, and the likelihood of recommending their program to a friend or colleague."

22-ii) Highlight unanswered new questions, suggest future research

Highlight unanswered new questions, suggest future research.

| | 1 | 2 | 3 | 4 | 5 | |
|---|---|---|---|---|---|---|
| subitem not at all important | ○ | ○ | ● | ○ | ○ | essential |

Clear selection

Does your paper address subitem 22-ii?

Copy and paste relevant sections from the manuscript (include quotes in quotation marks "like this" to indicate direct quotes from your manuscript), or elaborate on this item by providing additional information not in the ms, or briefly explain why the item is not applicable/relevant for your study

"As the data narrative for rising digital smoking cessation programs unfolds, areas ripe for future assessment include longer-term durability data, evaluation of the contributions to program engagement and abstinence rates of individual app functions such as coaching and breath sensor result tracking, and assessment of the cost-effectiveness of digital app-based interventions."

20) Trial limitations, addressing sources of potential bias, imprecision, and, if relevant, multiplicity of analyses

20-i) Typical limitations in ehealth trials

Typical limitations in ehealth trials: Participants in ehealth trials are rarely blinded. Ehealth trials often look at a multiplicity of outcomes, increasing risk for a Type I error. Discuss biases due to non-use of the intervention/usability issues, biases through informed consent procedures, unexpected events.

| | 1 | 2 | 3 | 4 | 5 | |
|---|---|---|---|---|---|---|
| subitem not at all important | ○ | ○ | ◉ | ○ | ○ | essential |

Clear selection

Does your paper address subitem 20-i? *

Copy and paste relevant sections from the manuscript (include quotes in quotation marks "like this" to indicate direct quotes from your manuscript), or elaborate on this item by providing additional information not in the ms, or briefly explain why the item is not applicable/relevant for your study

We acknowledge the aforementioned limitations. There was no blinding and the participants were informed of the possible tools available during the informed consent process. We felt there was no adequate way to blind the study as, obtaining informed consent required detailing the possible tools available and the sponsor of the study. The secondary outcomes are not adjusted for the multiple outcomes reported. Participants are not forced to use the app, coaching, NRT, and/or breath sensor. The direct effect of each program component is beyond the scope of this study as each component would warrant a separate clinical study as the primary outcome of interest. In addition, there may be additive or antagonistic interactions between the different modalities and favorable or unfavorable biases caused by the use or non-use of the each tool.

We felt it more important to see if the holistic program available by Pivot would engage participants more as documented by their self reported app use. This was a conscious choice made to compare the multi-modality pivot program plus NRT availability to a freely available standard of care app with NRT availability.

21) Generalisability (external validity, applicability) of the trial findings

NPT: External validity of the trial findings according to the intervention, comparators, patients, and care providers or centers involved in the trial

21-i) Generalizability to other populations

Generalizability to other populations: In particular, discuss generalizability to a general Internet population, outside of a RCT setting, and general patient population, including applicability of the study results for other organizations

| | 1 | 2 | 3 | 4 | 5 | |
|---|---|---|---|---|---|---|
| subitem not at all important | ○ | ○ | ○ | ● | ○ | essential |

Clear selection

Does your paper address subitem 21-i?

Copy and paste relevant sections from the manuscript (include quotes in quotation marks "like this" to indicate direct quotes from your manuscript), or elaborate on this item by providing additional information not in the ms, or briefly explain why the item is not applicable/relevant for your study

"This study also had several limitations. First, the inclusion criterion of intention to quit in the next 30 days resulted in a study population that may not reflect the general population of smokers. Aggregating across studies and populations, Prochaska et al. estimate that at any given time, approximately 20% of smokers are thinking of quitting smoking in the next 30 days, 35% to 40% are thinking of quitting in the next 6 months, and 40% to 45% are not seriously thinking of quitting [53]..."

21-ii) Discuss if there were elements in the RCT that would be different in a routine application setting

Discuss if there were elements in the RCT that would be different in a routine application setting (e.g., prompts/reminders, more human involvement, training sessions or other co-interventions) and what impact the omission of these elements could have on use, adoption, or outcomes if the intervention is applied outside of a RCT setting.

| | 1 | 2 | 3 | 4 | 5 | |
|---|---|---|---|---|---|---|
| subitem not at all important | ○ | ○ | ◉ | ○ | ○ | essential |

Clear selection

Does your paper address subitem 21-ii?

Copy and paste relevant sections from the manuscript (include quotes in quotation marks "like this" to indicate direct quotes from your manuscript), or elaborate on this item by providing additional information not in the ms, or briefly explain why the item is not applicable/relevant for your study

In a routine application setting, there would not be the videoconference biovalidation visits and the emailed online questionnaires. There were also more human-derived prompts in this study (6 reminder emails sent every other week from weeks 1-12 from the clinical team email) that would not be present in a non-study setting. And there would not be the screening phone call with study staff to enter the study. Overall, while human contact was low in this study, it was greater than what is present in a non-study setting, which could have increased adoption/use of the programs than might be seen in a routine application setting.

OTHER INFORMATION

23) Registration number and name of trial registry

Does your paper address CONSORT subitem 23? *

Copy and paste relevant sections from the manuscript (include quotes in quotation marks "like this" to indicate direct quotes from your manuscript), or elaborate on this item by providing additional information not in the ms, or briefly explain why the item is not applicable/relevant for your study

Clinicaltrials.gov NCT04955639; https://clinicaltrials.gov/ct2/show/NCT04955639

24) Where the full trial protocol can be accessed, if available

Does your paper address CONSORT subitem 24? *

Cite a Multimedia Appendix, other reference, or copy and paste relevant sections from the manuscript (include quotes in quotation marks "like this" to indicate direct quotes from your manuscript), or elaborate on this item by providing additional information not in the ms, or briefly explain why the item is not applicable/relevant for your study

The full protocol is not published but a summary of the protocol is available at Clinicaltrials.gov NCT04955639; https://clinicaltrials.gov/ct2/show/NCT04955639

25) Sources of funding and other support (such as supply of drugs), role of funders

Does your paper address CONSORT subitem 25? *

Copy and paste relevant sections from the manuscript (include quotes in quotation marks "like this" to indicate direct quotes from your manuscript), or elaborate on this item by providing additional information not in the ms, or briefly explain why the item is not applicable/relevant for your study

"Conflicts of Interest

JM, CF, MU, DB, and DU are current employees of Pivot Health Technologies Inc. (Pivot), the developer of the Pivot smoking cessation program. They receive salary and stock options from Pivot. DU is the President and CEO of Pivot and an investor in the company. JG is a paid statistical consultant."
The informed consent form notes,
"Funding:
This study is funded by Pivot Health Technologies Inc. (formerly Carrot Inc.). Pivot Health Technologies Inc. is the company that has developed one of the programs used in this study. Pivot Health Technologies Inc. develops products to help people quit smoking. You can learn more about the company at: https://my.pivot.co/ and https://pivot.co"

X27) Conflicts of Interest (not a CONSORT item)

X27-i) State the relation of the study team towards the system being evaluated

In addition to the usual declaration of interests (financial or otherwise), also state the relation of the study team towards the system being evaluated, i.e., state if the authors/evaluators are distinct from or identical with the developers/sponsors of the intervention.

| | 1 | 2 | 3 | 4 | 5 | |
|---|---|---|---|---|---|---|
| subitem not at all important | ○ | ○ | ○ | ○ | ○ | essential |

Does your paper address subitem X27-i?

Copy and paste relevant sections from the manuscript (include quotes in quotation marks "like this" to indicate direct quotes from your manuscript), or elaborate on this item by providing additional information not in the ms, or briefly explain why the item is not applicable/relevant for your study

Your answer

About the CONSORT EHEALTH checklist

As a result of using this checklist, did you make changes in your manuscript? *

- (•) yes, major changes
- ( ) yes, minor changes
- ( ) no

What were the most important changes you made as a result of using this checklist?

More extensive analyses for absolute and relative effect sizes, the handling of missing data with augmented imputation analyses for the primary endpoint, more extensive discussion of limitations

How much time did you spend on going through the checklist INCLUDING making changes in your manuscript *

5-7 days - we included new analyses that were complex

As a result of using this checklist, do you think your manuscript has improved? *

- (selected) yes
- no
- Other:

Would you like to become involved in the CONSORT EHEALTH group?

This would involve for example becoming involved in participating in a workshop and writing an "Explanation and Elaboration" document

- yes
- (selected) no
- Other:

Clear selection

Any other comments or questions on CONSORT EHEALTH

Your answer

STOP - Save this form as PDF before you click submit

To generate a record that you filled in this form, we recommend to generate a PDF of this page (on a Mac, simply select "print" and then select "print as PDF") before you submit it.

When you submit your (revised) paper to JMIR, please upload the PDF as supplementary file.

Don't worry if some text in the textboxes is cut off, as we still have the complete information in our database. Thank you!

Final step: Click submit !

Click submit so we have your answers in our database!

Submit

Clear form

Never submit passwords through Google Forms.

This form was created outside of your domain. Report Abuse - Terms of Service - Privacy Policy

Google Forms
